# A long journey towards genome editing technologies in plants: a technical and critical review of genome editing technologies

**DOI:** 10.3389/fgeed.2025.1663352

**Published:** 2025-11-11

**Authors:** Dylan Gallo, Anne-Cécile Meunier, Christophe Périn

**Affiliations:** UMR AGAP Institut, CIRAD, INRAE, Institut Agro, University Montpellier, Montpellier, France

**Keywords:** plant, genome editing, prime editing, SpCas9, knock-in

## Abstract

Advancements in genome editing technologies, notably CRISPR/Cas9, base editing (BE), and prime editing (PE), have revolutionized plant biotechnology, offering unprecedented precision in crop improvement to address the ongoing global warming challenge. This review provides a critical analysis of recent developments in SpCas9-based editing tools, emphasizing enhancements in editing efficiency and specificity and follow the chronological development of editing tools. We explore methodological innovations, including dual pegRNA strategies and site-specific integrases, that have expanded the potential of PE for precise gene insertions. By integrating insights into DNA repair mechanisms and leveraging SpCas9 enhancements, we outline future directions for the application of genome editing in plant breeding.

## Introduction

Since 2012, editing technologies can be used to introduce specific DNA modifications at specific sites in the genome. The interest of genome editing technologies such as base editing and prime editing for functional genomics and plant molecular breeding is obvious, as they can accelerate the introduction of specific beneficial alleles at target regions in plant genomes. Although there are numerous reviews that demonstrate the interest of these technologies for breeding and also provide lists of edited plants that get longer every year, to our knowledge there is no review that describes and critiques all these technical advances in a complete way, from SpCas9 to the recent development of prime editing. We have therefore chosen to describe and develop these improvements and advances since SpCas9. In fact, improving the efficiency and specificity of Base Editing (BE) and Prime Editing (PE) requires leveraging improvements made in native SpCas9 alongside technology-specific modifications, and *vice versa*, some improvements in BE and PE should also be critical to the efficiency of SpCas9. Therefore, we felt that an integrated view was important to maximize the future use of these technologies in plant breeding. This lengthy review, while following the chronological order of the development of editing technologies, focuses mainly on SpCas9 and to a lesser extent on orthologs to SpCas9. In the final section, we attempt to point the future of plant genome editing and the barriers that need to be overcome to realize its full potential in plant breeding.

## CRISPR/Cas9 and base editing: mechanisms and optimization

### CRISPR/Cas9 in a nutshell

#### CRISPR/cas systems: from evolutionary immunity to the genome-editing revolution

**FIGURE 1:**
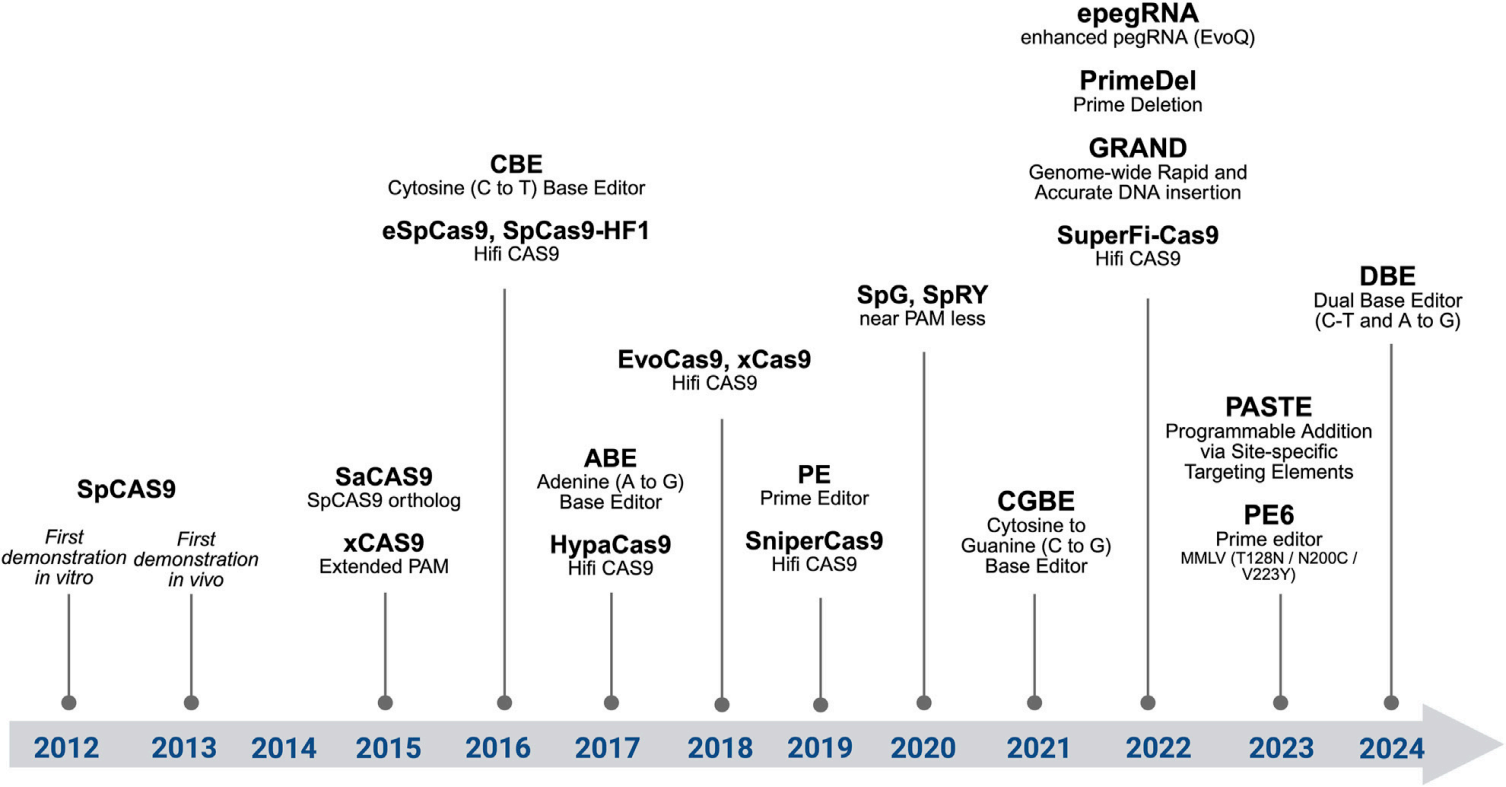
Chronological overview of major genome editing innovations. From SpCas9, high-fidelity variants, SpCas9 orthologs, base editors, prime editors, and advanced strategies (dual pegRNAs, PASTE).

The CRISPR/Cas9 system is an RNA-guided adaptative immune system in prokaryotes that targets foreign DNA where CRISPR/Cas stands for clustered regularly interspaced short palindromic repeats associated with Cas nuclease. This system emerged during the evolution of archaea and bacteria to prevent the invasion of these organisms by viruses (Barrangou et al., 2007; Jinek et al., 2012; Chylinski et al., 2014). There are two classes and 6 types of CRISPR/Cas systems known to date. Class 1 has effector modules composed of multiple Cas proteins, whereas the class 2 CRISPR mechanism requires a single Cas protein (CRISPR-associated protein) (Jinek et al., 2012; Chylinski et al., 2014). In class 2, Cas9 and Cas12 are DNA nucleases, whereas Cas13 is an RNA nuclease. In this review, we focus mainly on the widely used Cas9-based system; for further information about other CRISPR/Cas systems, see, for example, (Hille et al., 2018). See Figure 1 for a Chronological overview of major genome editing innovations.

In prokaryotes, the CRISPR repeat array is transcribed into a precursor RNA, which contains multiple CRISPR RNAs (crRNAs). Each of these crRNAs contains a single 20-base pair sequence that is complementary to invading DNA (Mojica et al., 2009; Gasiunas et al., 2012; Jinek et al., 2012; Chylinski et al., 2014), and repeats of conserved sequences that are complementary to a section of a transactivating CRISPR RNA called tracrRNA. The primary transcript is then processed into individual crRNAs by ribonuclease III (RNase III). The crRNA-tracrRNA complex interacts with the Cas9 protein to form an active RNA-guided nuclease. A protospacer adjacent motif (PAM) sequence, NGG, where “N” is A, T, C or G, is required for the binding of the Cas9 protein to a target sequence complementary to the spacer sequence.

The PAM acts as a sequence that distinguishes “self” from “non-self”, and PAMs are absent from bacterial chromosome targets (Mojica et al., 2009). Cas9 endonuclease cleaves the target DNA (Barrangou et al., 2007; Gasiunas et al., 2012; Jinek et al., 2012) adjacent to PAM sequence in this case called protospacer. Alternative type II systems (Chylinski et al., 2014), such as Cas12a (previously known as CPF1), recognize a different PAM sequence, i.e., TTTV, where “V” is A, C, or G, and induce double-strand breaks with cohesive ends (Zetsche et al., 2015; Safari et al., 2019; Alok et al., 2020). Other Cas proteins identified subsequently also recognize alternative PAMs (Shah et al., 2013; Leenay et al., 2016). Among type II proteins, *Streptococcus pyogenes* Cas9 (SpCas9) has been extensively used and modified for biotechnological applications.

#### SpCas9 ribonucleoprotein (RNP) complex formation

##### The SpCas9 endonuclease

**FIGURE 2:**
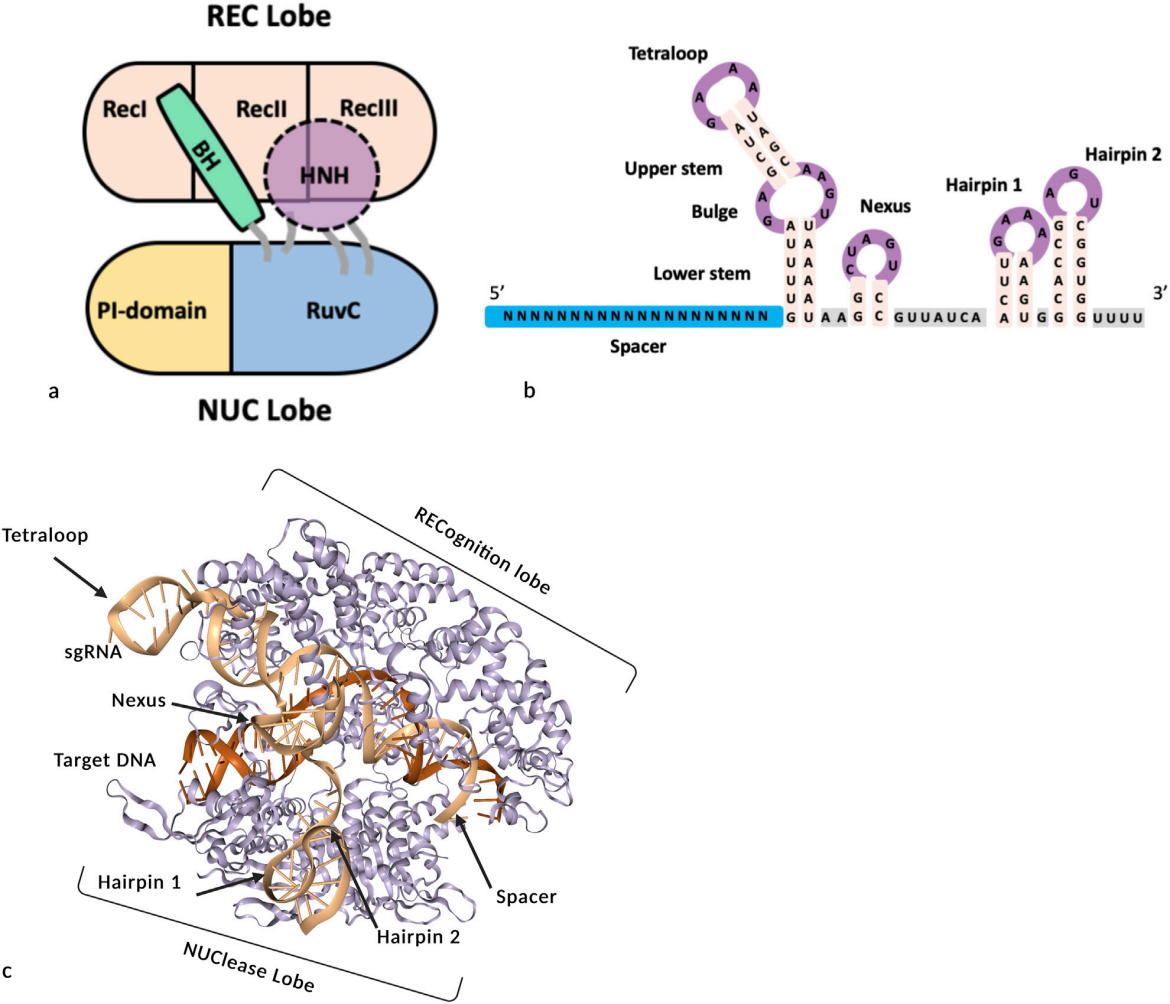
Schematic representations of SpCas9, sgRNA and the SpCas9/sgRNA/DNA complex. **(a)** Diagram of the main structural domains of SpCas9 domains, inspired by (Nishimasu et al., 2014; Dong et al., 2022). **(b)** Structural representation of the sgRNA. **(c)** 3D model of the SpCas9/target DNA/sgRNA complex generated with WebGL (4UN3, ProteinDataBank).

SpCas9 is a protein with 7 structural domains: REC1, REC2, REC3, BH (bridge helix), Pi (PAM interaction), HNH and RuvC (Jinek et al., 2012; Anders et al., 2014; Nishimasu et al., 2014). Cas9 contains two catalytic sites: HNH, which cuts the DNA strand complementary to the sgRNA, and RuvC which cleaves the nontargeted DNA strand. The HNH and RuvC domains can be inactivated to create nickases (D10A or H840A) or deadCas9 (D10A and H840A). The HNH, RuvC and Pi domains are located in the NUC (nuclease) lobe. The REC1, REC2 and REC3 domains form the REC (recognition) lobe and correspond to multiple alpha-helical recognition domains that enable sgRNA binding to target DNA (Figure 2a) (Jinek et al., 2012; Anders et al., 2014; Nishimasu et al., 2014; Jiang et al., 2015; Jiang et al., 2016; Pacesa et al., 2022a). The REC3 domain is fused to the HNH domain, and when the REC lobe interacts with RNA and DNA, its conformation changes, positioning the HNH domain opposite to RuvC to activate the generation of DNA double-strand breaks (Nishimasu et al., 2014; Zuo and Liu, 2017; Palermo et al., 2018; Pacesa et al., 2022a). The bridge helix (BH), an arginine-rich sequence, serves as a structural connector between the REC and NUC lobes and is crucial for mediating conformational transitions during Cas9 activation (Nishimasu et al., 2014; Palermo et al., 2018; Babu et al., 2019). The Pi domain comprises two subdomains, namely, the TOPO domain (for topoisomerase II homology), which is named because of its structural similarity with topoisomerase II (Jinek et al., 2012; Anders et al., 2014; Nishimasu et al., 2014; Zhang et al., 2019), and the CTD (C-terminal domain), which is the larger subdomain. The Pi domain recognizes and engages the PAM sequence in a positively charged groove and confers specificity to PAM site recognition (Jinek et al., 2012; Anders et al., 2014; Nishimasu et al., 2014; Zhang et al., 2019; Wang et al., 2021).

##### Single guide RNA of S. pyogenes

The single guide RNA (sgRNA) is an engineered fusion between the crRNA (CRISPR RNA) and the tracrRNA (transactivating crRNA) of the original *S. pyogenes* system (Jinek et al., 2012) (Figure 2b). The sgRNA guides Cas9 to its DNA target by recognizing a complementary sequence next to a PAM motif, triggering structural rearrangements that result in double-strand break (DSB) (Figure 2c). At the 5′ extremity of the sgRNA, the first module is the spacer consisting of a sequence of 20 nucleotides that pairs with the complementary sequence (or protospacer) of the target (Figures 2b,c). For Cas9 to bind to the target locus, the complementary sequence must be followed by a PAM sequence (Jinek et al., 2012; Briner et al., 2014; Szczelkun et al., 2014; Sternberg et al., 2015; Mekler et al., 2017). The spacer sequence can be broken down into two parts: a PAM-distal part from nucleotide 1 to 13 and a PAM-proximal part called “the seed” from nucleotide 14 to 20. While mismatches in the PAM-proximal ‘seed’ region typically disrupt Cas9 binding, the distal portion can tolerate some variation, though four mismatches can eliminate editing activity in plant cells (Modrzejewski et al., 2020). This phenomenon is the cause of off-target cleavage (Ivanov et al., 2020; Pacesa et al., 2022b).

The spacer is followed by the constant part, tracrRNA, which allows binding to the Cas9 protein. This area is composed of 6 distinct structures: the lower stem, the bulge and the upper stem, which compose the synthetic tetraloop, and the nexus, the linker and the two hairpin structures of the 3′ end (Anders et al., 2014; Briner et al., 2014; Nishimasu et al., 2014). In the tetraloop, the lower stem is required for the catalytic activity of Cas9 (Anders et al., 2014; Briner et al., 2014; Nishimasu et al., 2014). The bulge is an essential element, and even small modifications to its sequence or structure render the Cas9 complex inoperable (Briner et al., 2014). The upper stem in the sgRNA version has no essential role in the formation of the complex with the enzyme, as it does not exist in the original form. Its role is dispensable, but lengthening of this region by 5 bp slightly improves binding to the enzyme and therefore Cas9 efficiency (Dang et al., 2015).

The nexus module is described as the ‘core’ of the Cas9/sgRNA interaction and it also indirectly interacts with the target DNA strand (Figures 2b,c). This module is essential for the proper functioning of the sgRNA/Cas9 pair, as it has the most conserved nucleotide sequence of all of the modules in tracrRNA (Briner et al., 2014). The last two modules are hairpins 1 and 2. They consist of two stem‒loop hairpin structures that recognize and bind to Cas9 via interactions with the NUC lobe domain (Jinek et al., 2014; Nishimasu et al., 2014; Babu et al., 2021; Pacesa et al., 2022b). Although Hairpin 1 is not strictly required, its deletion greatly decreases cleavage efficiency (Briner et al., 2014), while hairpin 2 seems to be an essential structure (Briner et al., 2014). Hairpin 1 and the tetraloop, which emerged from Cas9 (Figure 2c), have been extensively used to add new secondary structures without compromising the efficiency of the complex (Riesenberg et al., 2022) to develop derived applications (Figure 2c).

### Evolution and improvements of SpCas9

#### Improving the gene editing specificity of SpCas9

**FIGURE 3:**
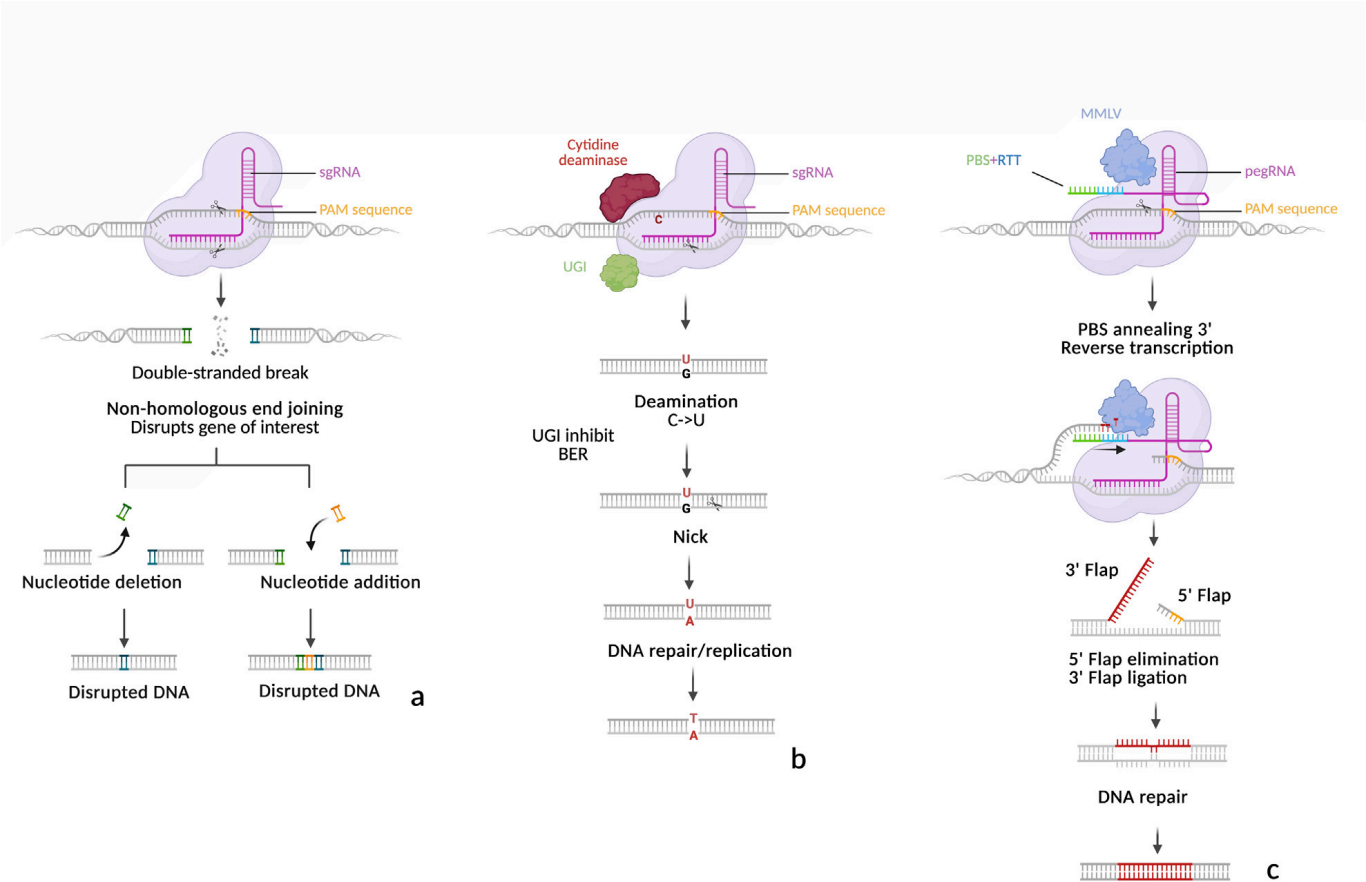
Schematic representation of SpCas9, Cytosine Base editor (CBE) and Prime editor (PE). **(a)** SpCas9-induced mutations. Upon recognition of the targeted strand by the SpCas9-sgRNA complex, SpCas9 cuts both the target strand and non-target strands, 3 nucleotides upstream of the PAM. The resulting DSB is repaired by NHEJ, which may restore the native sequence or introduce mutations through base pair insertions or deletions. **(b)** Cytosine Base Editing (BE3). The cytosine deaminase converts a cytosine (C) to an uracil (U) on the non-target strand. UGI inhibits BER to stabilize the U, while the nickase (D10A) cleaves the target strand near the PAM, triggering MMR of the unedited strand. The guanine (G) is then replaced by adenine (A) and during replication, the U is interpreted as thymine (T). **(c)** Prime editing (PE2). After recognition of the target strand, the nickase (H840A) cuts the non-target strand near the PAM. The primer binding site (PBS) anneals the complementary ssDNA, allowing MMLV to reverse transcribe the RT template. Competition between the 5′ and 3′ ssDNA flaps follows. If the 3′ flap is retains, it can form a heteroduplex with the unedited strand. MMR may then resolve the heteroduplex, leading either to insertion of the edited sequence or restoration of the original DNA.

A major challenges of the development of this technology lies in reducing unintended DNA cleavage events, the off-target phenomena (Hsu et al., 2013; Tsai et al., 2015; Doench et al., 2016; Cameron et al., 2017; Lazzarotto et al., 2020; Pacesa et al., 2022b), to enhance genome editing specificity. Table 1 summarizes all of the GE specificity and efficiency approaches described in the following paragraphs. See also Figure 3a for a schematic view of the mode of action of the SpCas9 sgRNA complex.

**TABLE 1:** Summary of key improvements in the specificity and efficiency of CRISPR/Cas9 editing. See main text for references.

	Name	Description	Purpose	Plant species	References
GE efficiency					
SpCAS9	GE max	R221K and N394K mutations	DSB efficiency	NT	Spencer and Zhang (2017)
	TREX2	Fusion of a TREX2 exonuclease	Increase severity of mutation	AT, NB, OS	Liu et al. (2024a), Capdeville et al. (2023)
	BP NLS	Adding Bipartite NLS instead of single NLS	More efficient nucleus import of SpCAS9	NT	Develtere et al. (2024)
sgRNA	hpsgRNA	Adding hairpin sequence in 3'end of sgRNA	Linking between sgRNA, SpCAS9 and target sequence	NT	Dang et al. (2015)
	Hairpin 1 elongation	Elongating hairpin 1 until reaching a Tm of 71°	Increase probably sgRNA stability	NT	Riesenberg et al. (2022)
	dsgRNA	dead guides sequences	Increase editing in close chromatin regions	OS	Liu et al. (2019a)
	Composite	Composite promoter (RNApolII/RNApolIII)	Increase level of sgRNA transcription	NT	
GE specificity					
SpCAS9	eSpCAS9	Mutations in NUC lobe	High specificity, strong reduction of efficiency	AT, OS, GM	Vakulskas et al. (2018), Raitskin et al. (2019), Xu et al. (2019), He et al. (2022)
	HiFiCAS9, CAS9HF1, HypaCAS9	Mutations in REC3 domain	High specificity, strong reduction of efficiency	NT, NT, OS	Vakulskas et al. (2018), Kleinstiver et al. (2016), Chen et al. (2017), Xu et al. (2019)
	EvoCAS9	Mutations in REC1 and REC3 domains	High specificity, strong reduction of efficiency	NT	Casini et al. (2018)
	SpartaCAS	Mutations in REC1 and RuvC domains	High specificity, strong reduction of efficiency	NT	Cerchione et al. (2020)
	Sniper-CAS9, SuperFI-CAS9	Mutations in REC3, RuvC and HNH domains	High specificity, medium reduction of efficiency	NT, NT	Lee et al. (2018), Bravo et al. (2022)
	Sniper2L	E1007L mutation	High specificity without comprimising efficiency	NT	Kim et al. (2023)
	SpCAS9 VQR, EQR, VRER	D1135V/R1335Q/T1337R, D1135E/R1335Q/T1337R, D1135V/G1218R/R1335E/T1337R mutations	Alternative NGG PAM (NGA, NGAG, or NGCG)	OS, AT	Kleinstiver et al. (2015b), Hu et al. (2018b), Hua et al. (2019), Yamamoto et al. (2019)
	xCAS9	E480K/E543D/E1219V core mutations	Alternative NGG PAM (NG, GAA and GAT)	OS, AT	Hu et al. (2018a) Raitskin et al. (2019), Zhong et al. (2019)
	CAS9-NG	Near PAMless	NG	OS, AT, GM	Nishimasu et al. (2018), Hua et al. (2019), Zhang et al. (2020), Zeng et al. (2020)
	SpRY, SpG	Near PAMless	NRN (R = A or G) and NAN; NGA, NGG, NGT	OS	Walton et al. (2020), Li et al. (2021), Xu et al. (2021)
	SpCAS9-VP64	Fusion with a transcriptionnal activator	Increase editing in close chromatin regions	OS	Liu et al. (2019a)
sgRNA	CRISPOR/TEFOR	Online software to design sgRNA	Choose high efficient and high specific sgRNA for a target	—	Haeussler et al. (2016), Concordet and Haeussler (2018)
	dsgRNA/hpsgRNA	Adding dead or truncated guides	Reduce/suppress off target by masking non specific target	NT	Kocak et al. (2019)
	hpsgRNA	Adding hairpin sequence in 3'end of sgRNA	Reduce off target by limiting unspecific Rloop formation	NT	Dang et al. (2015)

NT, not tested in plants; OS, Rice; AT, A. thaliana; N, Nicotiana benthamiana; GM, Glycine max.

##### High-fidelity SpCas9

To improve specificity and reduce off-target effects, several high-fidelity (HiFi) SpCas9 variants have been developed, often with reduced cleavage efficiency as a trade-off. These include eSpCas9 (Vakulskas et al., 2018), Cas9HF1 (Kleinstiver et al., 2016), HypaCas9 (Chen et al., 2017), HiFiCas9 (Vakulskas et al., 2018), EvoCas9 (Casini et al., 2018), SpartaCas (Cerchione et al., 2020), Sniper-Cas9 (Lee et al., 2018), SuperFi-Cas9 (Bravo et al., 2022), and Sniper2L (Kim et al., 2023). Among them, Sniper2L appears to offer the best balance, significantly increasing specificity without major loss in activity, due to targeted mutations in the RuvC region involved in mismatch recognition (Bravo et al., 2022; Kim et al., 2023). In plant cells, while SpCas9HF2 has no editing capacity and HypaCas9 has a 50% reduction in editing efficiency compared with SpCas9 (Xu et al., 2019), eSpCas9 has comparable or greater efficiency and increased specificity up to 20-fold (Raitskin et al., 2019; Xu et al., 2019; He et al., 2022) in rice, soybean and *Arabidopsis thaliana.* Thus, eSpCas9 emerges as a promising tool for crop genome editing when minimizing off-target control is an important issue, whereas the usefulness in plants of other high-fidelity SpCas9s, such as Sniper2L Cas9, that currently offers the best specificity and cleavage efficiency, equivalent to that of SpCas9 in mammalian cells (Kim et al., 2023), remains to be demonstrated.

Engineering Cas9 to recognize other PAMs is a key strategy devised to address specificity issues when no specific guides are available with SpCas9. SpCas9 derivatives, such as Cas9-VQR, with D1135V/R1335Q/T1337R mutations; Cas9-EQR, with D1135E/R1335Q/T1337R mutations; and Cas9-VRER, with D1135V/G1218R/R1335E/T1337R mutations, have been engineered to recognize non-canonical PAMs, expanding the targeting scope from NGG to sequences such as NGA, NGAG and/or NGCG (Kleinstiver et al., 2015b) (Table 1). These extended variants offer a wider choice of sites to target, an advantage when no efficient and/or specific sgRNA can be used with spCas9. Similarly, non-NG Cas9s (Miller et al., 2020) or ‘near-PAMless’ versions such as Cas9-NG (Nishimasu et al., 2018), SpG, and SpRY (Walton et al., 2020) have extended this capacity by relaxing the NGG PAM restriction to NGN or even more complex motifs such as NRN or NYN. Notably, discoveries surrounding xCas9, a new Cas9 variant that emerged from protocols for phage-assisted evolution, have led to substantial progress in this area (Hu J. H. et al., 2018). xCas9s recognize an extended array of PAM motifs, such as NG, GAA, and GAT, thereby providing broader targeting compatibility (Hu J. H. et al., 2018). They enhance specificity while maintaining the cleavage efficacy of SpCas9 in mammalian cells, particularly the models xCas9 3.6 and xCas9 3.7 (Hu J. H. et al., 2018). However, broader PAM compatibility can pose new challenges in off-target control, as the number of binding sites in a genome increase significantly. Cas9 variants that recognize alternative PAMs, including xCas9, Cas9-VQR, Cas9-EQR and Cas9-NG, have been successfully developed in plants but have a cleavage efficiency often lower than that of SpCas9 [see, for example, (Hu X. et al., 2018; Hua et al., 2019; Yamamoto et al., 2019; Zhong et al., 2019)]. Among these, xCas9, was described as having similar (Raitskin et al., 2019) or higher editing efficiency (Zhong et al., 2019) but better specificity than SpCas9 in *A. thaliana* and rice. Moreover, xCas9 and high-fidelity exCas9 seem to significantly improve the specificity while maintaining the efficiency (He et al., 2022). Near-PAMless versions, CAS9-NG (Hua et al., 2019; Zeng et al., 2020; Zhang et al., 2020) and SpRY and SpG were also active in plant (Li et al., 2021; Xu et al., 2021).

##### High-fidelity sgRNAs

Software packages such as CRISPOR (Haeussler et al., 2016; Concordet and Haeussler, 2018), have been created to predict potential sgRNAs for genome editing, assessing both their on-target efficiency and off-target risk for sequenced genomes. Despite advancements, Off-target prediction software can miss some sites, especially when the reference genome is missing, incomplete or poorly annotated. On the other hand, the performance of these software programs is improving, particularly with the development of model prediction algorithms or artificial intelligence (AI)-based software (Pacesa et al., 2022a; Dixit et al., 2023).

One approach to limiting off-target activity involves co-delivering additional guides during editing, either catalytically inactive or truncated, that still bind but do not cleave DNA, shielding off-target loci (Fu et al., 2014; Coelho et al., 2020). The first functional guide targets the editing zone, and the other guides mask the off-target sites (Fu et al., 2014; Coelho et al., 2020; Rose et al., 2020). This makes it possible to use a guide that is not very specific but is necessary to induce a specific mutation while limiting the formation of off-target mutations. This approach has one drawback: if the number of predicted off-target effects is high, many dead/truncated guides need to be multiplexed.

Incorporating a 3′ hairpin structure into the sgRNA to form hpsgRNA (Kocak et al., 2019) has been shown to increase the specificity of the complex for different Cas9s and different targets without significantly reducing the efficiency of editing (Kocak et al., 2019). This approach was found to be superior to the strategy using truncated RNA (Kocak et al., 2019). Other modifications involve the addition of a hairpin structure, which likely stabilizes sgRNAs and thus reduces their turnover by increasing their availability for binding to SpCas9. Although these approaches using high-fidelity sgRNAs to improve editing specificity are promising, to the best of our knowledge they have not yet been reported in plant systems.

#### Improving the gene editing efficiency of SpCas9

##### High-efficiency SpCas9

The DSBs generated by SpCas9 have blunt or slightly staggered ends (Longo et al., 2024), which are processed mainly through the classical nonhomologous end joining (cNHEJ) repair system (Gehrke et al., 2022). Most DSBs are thus repaired until the appearance of random mutations induced by cNHEJ errors (Gehrke et al., 2022), and these repeated cuts are also responsible for translocation and chromosomal rearrangement (Yin et al., 2022). Coexpressing TREX2 with 3′-5′ exonuclease activity (Certo et al., 2012), which is involved in the DNA repair system (Ko et al., 2020), increases the mutation rate by degrading these overhang breaks and leads to the fixation of deletion-type mutations (Certo et al., 2012). Fusing SpCas9 to TREX2 exonuclease significantly increases editing efficiency while strongly inhibits chromosomal rearrangement (Yin et al., 2022). In plants, RNA viruses are used to co-deliver sgRNA and TREX2 (Liu D. et al., 2024) and increase only the mutation rate, i.e., the editing efficiency (Liu D. et al., 2024). Similarly, the recruitment of TREX2 to the SUNTAG system increases the mutation and deletion rates in *A. thaliana* by a factor of two (Capdeville et al., 2023). A mutation screen identified the combination of the R221K and N394K mutations in SpCas9 as enhancing editing activity twofold for eight targets, likely by facilitating HNH alignment during cleavage (Spencer and Zhang, 2017) in human cells. Finally, the use of a strong promoter to increase the expression of SpCas9 together with a bipartite NLS increased the editing rate (Develtere et al., 2024).

##### High-efficiency sgRNAs

A higher efficiency of GE is achieved with a spacer GC content of approximately 40%–60% (Liu et al., 2016; Malik et al., 2021), and it is recommended that the GC content of the PAM-proximal region do not exceed 50% (Malik et al., 2021) and that of the PAM-distal region be more than 50% (Labuhn et al., 2018). Poly-T stretches within sgRNA can trigger RNA polymerase III stalling or backtracking and should be avoided (Nielsen et al., 2013). Hairpin RNA aptamers are sometimes added for GE and increase sgRNA efficiency, but adding more than two aptamers in either the upper stem or hairpin reduces cleavage efficiency (Dong et al., 2022). The cleavage efficiency can be significantly increased by extending the upper stem of the tetraloop by up to five base pairs (Dang et al., 2015), and by elongating hairpin 1 to achieve a melting temperature (Tm) of 71 °C (Riesenberg et al., 2022). These strategies will be of particular interest to test in plant systems. Intra molecular interactions between the spacer and tracrRNA or crRNA end can interfere with Cas9 activity and reduces cleavage efficiency.

GE efficiency rates vary according to chromatin opening in human cells (Chen et al., 2016; Daer et al., 2017) and in rice (Liu G. et al., 2019). On average, they are higher in open regions than in closed regions, and reversing a closed chromatin state to an open state restores GE efficiency (Chen et al., 2016; Daer et al., 2017). The presence of nucleosomes directly inhibits Cas9 binding and cleavage *in vitro* and *in vivo* (Horlbeck et al., 2016). By using additional dead sgRNAs close to the GE target region, it is possible to increase GE levels in rice (Liu G. et al., 2019), and interestingly, even in open chromatin regions, this strategy increases GE levels (Liu G. et al., 2019). Finally, the use of a version of SpCas9 fused to a transcriptional activator, SpCas9-VP64, also increases GE levels in closed chromatin regions, and combinatorial strategies, e.g., the use of dsgRNA and a transcriptional activator, have a synergistic effect (Liu G. et al., 2019).

##### Multiplexing sgRNA expression

Optimizing multiplex sgRNA expression is essential for plant breeding, with idea of simultaneously introducing multiple agronomically important alleles, to speed up varietal development. Currently, five distinct systems have been used for multiplexing sgRNA expression [79]. In the first system, the sgRNAs can be expressed under the control of their independent promoters (Ma et al., 2015). In the other systems, crRNAs or sgRNAs are under the control of a single promoter, and the polycistronic sequence is then posttranscriptionally cleaved. Each sgRNA can be separated with 5′and 3′tRNA sequences recognized by endogenous RNAse P and Z (Xie et al., 2015) or with 5′ HH (hammerhead) and 3′ HDV (hepatitis delta virus) ribozymes (Tang et al., 2016; Zhang et al., 2021) or by a CSY4 hairpin recognized by a coexpressed CSY4 RNA endonuclease (CRISPR/Cas9 subtype Ypest protein 4) (Cermak et al., 2017).

A study focusing on Cas12a compared the efficiency of various multiplexing strategies, and while the findings are specific to Cas12a, they may offer insights applicable to SpCas9 systems (Zhang et al., 2021). The use of an RNA pol II (or composite) promoter is important for efficient multiplexing efficiency, as RNA pol III promoters like U6 and U3 may have limitations in transcribing longer RNAs. The best multiplexing system uses HH and HDV ribozymes at the 5′and 3′ends, respectively, to separate each sgRNA. In this system, mutations were obtained in 15 out of 16 targets across seven primary transformants, with one plant having mutations in all targeted loci (Zhang et al., 2021). Another study evaluated these multiplexing systems in the context of prime editing and found the CSY4-based system to be the most efficient (Ni et al., 2023). Therefore, conclusions on the optimal multiplexing system remains premature, as performance may vary depending on the specific Cas nuclease employed.

#### Cas9 orthologs as alternatives to SpCas9

There are many orthologs to SpCas9 from different prokaryotic organisms that have been used in plants, such as SaCas9 (*Staphylococcus aureus* Cas9) (Steinert et al., 2015), iSpyMacCas9, a hybrid between the PAM interacting (PI) domain of SpCas9 and the PI domain of Cas9 SmacCas9 (*Streptococcus macacae Cas9)* (Sretenovic et al., 2021b), St1Cas9 (*Streptococcus thermophilus Cas9*) (Steinert et al., 2015), Nm1Cas9 and Nm2Cas9 (*Neisseria meningitidis Cas9*) (Xu R. et al., 2022) or ScCas9 (*Streptococcus canis Cas9*) (Xu et al., 2020b). They offer certain advantages over SpCas9, such as recognition of alternative PAMs (Table 2), but have variable efficiencies and fidelity. SaCas9 is the most interesting alternative to SpCas9 in plant genome editing, with comparable or even superior editing efficiency than SpCas9 in many plant species (Steinert et al., 2015; Kaya et al., 2016; Jia et al., 2017; Qin et al., 2019; Zhang et al., 2022). Unlike SpCas9, SaCas9 recognizes a more specific PAM sequence (5′-NNGRRT-3′) which may limit the range of editing target regions. To broaden the number of targetable sites, the SaCas9-KKH variant, incorporating E782K/N968K/R105H mutations, recognizes an expanded PAM (5′-NNNRRT-3′) (Kleinstiver et al., 2015a) and appears to be as effective as the original nuclease in rice (Qin et al., 2019). Additionally, a high-fidelity variant of SaCas9 (N260D mutation) has been developed to minimize off-targets but has not yet been used in plants (Xie et al., 2020).

**TABLE 2:** Summary of SpCas9 orthologs used in plants and their main characteristics.

	PAM	Efficiency	Specificity	Remarks	Plant species	References
CAS9 orthologs						
SpCas9	5’-NGG-3’	High	Medium			
SaCas9	5’-NNGRRT-3’	Medium to high	Medium	Best alternative to SpCAS9	AT, OS, CS, GM	Steinert et al. (2015), Kaya et al. (2016), Jia et al. (2017), Qin et al. (2019), Zhang et al. (2022)
eSaCAS9	5’-NNGRRT-3’	Medium to high	High	N260D mutation	NT	
SaCAS9-KHH	5’-NNNRRT-3’	Medium to high	Medium	E782K/N968K/R105H mutations	OS	Kleinstiver et al. (2015a), Qin et al. (2019)
ScCAS9	5' -NNG- 3'	High^a^	Not tested	PAM 5' -NAG- 3' in rice	OS	Xu et al. (2020b)
St1CAS9	5' -NNAGAAW- 3'	Medium^a^	Not tested		AT	Steinert et al. (2015)
iSpyMacCAS9	5' -NAA- 3'	Medium^a^	Not tested	PAM 5'-NAAR- 3' in rice	SL, OS, PT	Sretenovic et al. (2021b)
Nm1Cas9 and Nm2Cas9	5' -NNNNGATT- 3' 5' -NNNNCC- 3'	High^a^ (Nm1CAS9) Low to medium^a^ (Nm2CAS9)	Medium^a^	S593Q/W596R mutations in Nm2CAS9 increase editing efficiency	OS	Xu et al. (2022a)

^a^ Except for SaCas9, AsCas12a and LbCas12a data in plants are limited. Efficiency and specificity assesments should therefore be interpreted with caution.

NT, not tested in plants; OS, Rice; AT, A. thaliana; GM, Glycine max; PT, Populus trichocarpa; SL, Solanum lycopersicum; CS, Citrus sinensis

#### Knock-in by NHEJ/HDR

Knock-in consists of introducing complex modifications such as, for instance, HA tags, introduction of a reporter gene such as GFP, insertion of an enhancer into a promoter. The use of SpCas9 has significantly advanced targeted insertion, by enabling precise genome editing coupled with the activation of cellular repair systems such as NHEJ and HDR (homologous DNA repair), leading to insertion.

The NHEJ-KI technique in plants requires codelivery of SpCas9 complex, which targets the inserted zone, and a DNA template to be inserted (ssDNA oligonucleotides, dsDNA, plasmids, PCR products, etc.). Efficient insertion requires the simultaneous delivery of a large quantity of donor DNA with SpCas9 to reduce indel formation and increase the likehood of template’s presence near the DSB site. Bringing the matrix to be inserted close to the target also improves the insertion rates (Aird et al., 2018; Ali et al., 2020).

Lu et al. used this approach to insert tags into the rice genome (Lu et al., 2020). They first reported that it was possible to insert tags using dsDNA oligos but not ssDNA. Modification of oligonucleotides at the 5′end by phosphorylation to promote NHEJ and at the 5′and 3′ends by phosphorothioate linkage to protect against endogenous exonucleases strongly improved KI rates. Using 60-bp tags, they achieved insertion efficiencies of approximately 25%, i.e., a 5–6-fold improvement over unmodified oligonucleotides for several targets (Lu et al., 2020). To test the insertion of larger fragments, they produced matrices for insertion via PCR of fragments with protected oligonucleotides (Lu et al., 2020). The efficiency decreased with increasing size of the inserted fragment (5% with 2 kb), and the rate of deletions at the junction increased significantly, probably because only one strand of the PCR products was protected, in contrast with oligonucleotides protected on both strands.

Similar strategies, without end protection, were developed, and fragments of several kilobases were successfully inserted, with efficiency rates of 2%–3%, suggesting that it is indeed possible to insert long fragments by biolistic techniques via NHEJ but at the cost of low efficiency (Li et al., 2016; Xu et al., 2020c). Finally, to achieve seamless insertions and sequence replacements, Lu et al. introduced the tandem repeat HDR (TDR-HDR) method, which combines initial NHEJ-mediated insertion of a first sequence followed by HDR-recombination facilitated by a second sRNA. The second sgRNA is used to cleave the sequence at the first inserted oligonucleotide, stimulating recombination via HDR between the two homologous fragments (Lu et al., 2020). This technique can be used to insert any sequence with an efficiency of approximately 15%.

While effective for sequence insertion or replacement, biolistics methods as opposed to *Agrobacterium tumefaciens* delivery, raises known problems, including extensive genomic rearrangements (deletions, duplications) and multiple inserted transgenes (see, for example, (Liu J. et al., 2019; Banakar et al., 2019), for a discussion of biolistic drawbacks). Currently, most technological developments for knock-in revolve around the use of prime editing (PE) and dual pegRNA.

### Base editing (BE): transitions and transversions without DSBs

Base editor 1 (BE1), the inaugural base editor, was engineered by fusing a catalytically inactive Cas9 (dCas9) with the rat cytidine deaminase rAPOBEC1 (Komor et al., 2016). The cytidine deaminase targets ssDNA within the R-loop formed by the dsDNA-sgRNA-dCas9 complex, converting cytosine (C) to uracil (U) within a limited editing window. During replication, the Mismatch Repair (MMR) system may replace the guanine (G) opposite the uracil (U) with adenine (A), and the uracil is converted to thymine (T), resulting in a C·G to T·A transition. However, BE1’s editing efficiency was limited by repair cellular mechanisms such as uracil excision by Uracil DNA glycosylase (UDG) and the mismatch repair pathway favoring restoration of the original base pair. Uracil DNA glycosylase (UDG) recognizes and excises U through the base excision repair (BER) pathway, either restoring the original C·G pair or introducing unintended mutations. Mismatch repair does not always favor the desired mutation, leading to reversions. To counteract UDG-mediated excision, BE2 was developed by adding a uracil glycosylase inhibitor (UGI) from the *Bacillus subtilis* phage PBS1, which significantly improved editing efficiency (Komor et al., 2016). Finally, BE3 introduced a key improvement: dCas9 was replaced with a nCas9 (D10A), introducing a nick in the non-edited strand to bias repair toward the edited strand (Komor et al., 2016). See Figure 3b for a schematic view of the mode of action of a CBE. This modification significantly increased the frequency of permanent C·G to T·A conversions. Subsequent cytosine base editors (CBEs) have incorporated both nCas9 and UGI to maintain high editing efficiency (Kim et al., 2017). The introduction of BE4 and BE4max, which incorporate a dual UGI system, further enhanced UDG inhibition and improved editing efficiency (Komor et al., 2017; Koblan et al., 2018).

Following the advent of CBE(s), adenine base editors (ABEs) were quickly developed by fusing a nCas9 with a synthetic tRNA adenosine deaminase (Gaudelli et al., 2017). Unlike CBEs, ABEs catalyze A·T to G·C conversions, rather than C·G to T·A. Adenine deamination produces inosine (I), which is read as guanine (G) during DNA replication, eliminating the need of a UGI (Gaudelli et al., 2017). However, as with CBEs, the use of nCas9 (D10A) facilitates preferential repair of the edited strand, enhancing editing efficiency. For CBEs, the main cytidine deaminases used include rAPOBEC1 and AID/APOBEC3A (Wang et al., 2018), whereas ABEs rely on engineered adenosine deaminases (Gaudelli et al., 2017). A distinct category, C-to-G base editors (CGBEs), enables C·G to G·C transversions (Chen L. et al., 2021; Kurt et al., 2021). Unlike CBEs, which include a UGI to prevent uracil excision, CGBEs replace UGI with either UDG, also called eUNG [113] or BER pathway proteins [114]. UDG removes uracil (U), and under specific conditions, the repair machinery preferentially insert guanine (G) instead of cytosine (C), leading to a C-to-G conversion. More recently, dual base editors (DBEs), which combine ABEs and CBEs, have been developed to enable simultaneous C-to-T and A-to-G conversions within the same editing window (Fan et al., 2024; Ma et al., 2024). Such hybrid editors broaden the range of programmable base editing applications, especially in the context of multiplexed genetic modifications. The SWISS system use sgRNA scaffold (scRNA) embedded with two different aptamers, each binding to a specific protein fused to either a CBE or an ABE, enabling simultaneous base editing at two separate targets (Li C. et al., 2020). These technologies were rapidly applied to plant genome engineering, enabling precise genetic modifications, for CBE (Lu and Zhu, 2017; Zong et al., 2017; Kang et al., 2018), ABE (Hua et al., 2018; Li et al., 2018) and CGBE (Sretenovic et al., 2021a; Tian et al., 2022). For example, base editing has been used to enhance Nitrogen Use Efficiency in rice by introducing a modified NRT1.1B allele (Lu and Zhu, 2017).

To broaden the range of targetable loci, scientists engineered base editors utilizing Cas9 orthologs capable of recognizing alternative or expanded PAM sequences. SaCas9 (from *S. aureus* Cas9), recognizing the 5′ NNGRRT 3′ PAM motif, has been widely used due to its compact size and efficiency (Kleinstiver et al., 2015a; Qin et al., 2019). Further refinements included Cas9 variants with relaxed PAM requirements, such as SpCas9-NG, SpG, and SpRY, which enable broader target site selection (Kim et al., 2017; Hua et al., 2019). Additionally, high-fidelity Cas9 variants, including xCas9 and evoCas9, were engineered to reduce off-target activity while maintaining efficient base editing capabilities (Zhong et al., 2019; Zeng et al., 2020; Zhang et al., 2020).

Despite their advantages, CBEs introduce unintended mutations due to non-specific deamination, leading to random single-nucleotide variants independent of sgRNA targeting. Such off-targets effects have been documented in both in rice (Jin et al., 2019) and mouse embryos (Zuo et al., 2019; Lee et al., 2020). In contrast, ABEs do not exhibit the same genome-wide off-target effects (Jin et al., 2019; Lee et al., 2020). Additionally, both CBEs and ABEs have been reported to deaminate RNA, causing widespread transcriptome-wide RNA editing (Grunewald et al., 2019). To mitigate these issues, improved CBEs have been engineered with enhanced specificity and reduced off-target activity (Grunewald et al., 2019; Jin et al., 2020). An additional challenge is the reduced efficiency of BEs in dicotyledonous plants compared to monocots, possibly due to inadequate promoter strength in dicots (Kang et al., 2018; Niu et al., 2023). Furthermore, base editors are constrained by their editing window: CBEs typically edit cytosines within positions 4-8 from the 5′end of the spacer, ABEs operate within positions 4–7. When multiple cytosines reside within the CBE’s editing window (positions 4–8), simultaneous editing can occur, potentially comprising specificity. Strategies to narrow the editing window have been developed (Kim et al., 2017; Jiang et al., 2018), along with high-fidelity base editors that enhance precision (Zhong et al., 2019; Zeng et al., 2020; Zhang et al., 2020).

## Prime editing derived technologies: applications and innovations

Prime Editing (PE), introduced after the development of Base Editors (BEs), facilitates precise genome modifications beyond base substitutions. Unlike BEs, which only convert one base pair into another, PE can introduce targeted insertions, deletions (indels), and complex edits all without requiring a double-strand break (DSB) or a donor DNA template (Anzalone et al., 2019). Due to its greater versatility and reduced off-target effects, Prime Editing is now a major focus in genome editing research, offering a more precise and flexible alternative to traditional base editing even if last generation of base editors can achieved higher efficiency for transversion and conversions than prime editors.

### Prime editing (PE): a flexible tool for precise modifications

Prime editing represents a major advancement from CRISPR/Cas9 technology, offering precision beyond traditional genome editing tools. The prime editor is composed of an nCas9 (H840A or D10A) fused to the N-terminus of a reverse transcriptase, initially from Moloney murine leukemia virus (MMLV) and a modified sgRNA called pegRNA. This technology was introduced and validated in 2019 by Anzalone et al. (2019). The nCas9-MMLV complex binds to its target to form an R loop, after which nickase cleaves the nontarget strand (Figure 3c). The free 3′ hydroxyl end of the nontarget strand then binds to the PBS (primer binding site) of the pegRNA 3′extension. The MMLV then reverse transcribes the template (pegRNA 3′ extension) containing the mutation(s) to be introduced from the free DNA 3′ end, which has a priming function for the initiation of reverse transcription. Following removal of the 5′flap, the 3′flap can anneal to the target site, forming a DNA heteroduplex. In the original versions of prime editors, such as PE1 and PE2 (Anzalone et al., 2019), the heteroduplex is spontaneously resolved by MMR by returning either to the WT sequence or the sequence to be introduced. In the PE3 version (Anzalone et al., 2019), a second and classic guide is used to cleave the target strand at a short distance from the introduced mutations, favoring the introduction of the targeted mutation by MMR. This technology provides a broader range of flexibility than base editing making it more versatile for complex genome modifications. In theory, it can be used to introduce almost any mutation needed, ranging from a single modified base to more complex modifications (deletions, insertions, modifications of several bases, etc.). Initial attempts to apply PE in plants showed low efficiencies, typically under 1% and rarely above 10% (Li H. et al., 2020; Lin et al., 2020). Numerous improvements were soon published, enabling plant biologists to obtain rates increasingly close to those obtained in animal systems but also to improve specificity, defined as creating the desired allele while reducing or eliminating unwanted alleles. Recent studies have reported optimized plant prime editors capable of achieving over 20% efficiency for multi-nucleotide edits and small tag insertions in rice, with minimal indel formation (Li et al., 2023; Zhong et al., 2024). Table 3 summarizes all of the PE improvements described in the following paragraphs.

**TABLE 3:** Summary of major advancements in prime editing specificity and efficiency. See main text for references.

	Name	Description	Purpose	Plant species	References
PE efficiency					
nCAS9	PE max	R221K and N394K mutations	nCAS9 efficiency	OS, ZM, TA	Li et al. (2022a), Ni et al. (2023), Qiao et al. (2023)
	MLHd1, MutS	Fusion protein inhibiting the MMR pathway	Increase probability of keeping edited flap. Contradictory results in plants	OS	Chen et al. (2021b), Ferreira Da Silva et al. (2022), Li et al. (2022a), Vu et al. (2022), Liu et al. (2024c)
MMLV	△RNAseH	Deletion of RNAseH	Increased stabilization of the pegRNA/DNA heteroduplex	OS, TA	Doman et al. (2023), Zong et al. (2022), Ni et al. (2023)
	V223A	V223A mutation	Increased MMLV processivity	TA	Ni et al. (2023)
	NC	Fusion with the N terminus of a nucleocapsid protein		TA	Ni et al. (2023)
pegRNA	evopreQ1	Hairpin structure	3' End protection by pegRNA	OS, TA, ZM	Nelson et al. (2022) Jiang et al. (2022), Li et al. (2022a), Zong et al. (2022), Ni et al. (2023), Qiao et al. (2023)
	LITe	Random linker between the PBS and RT template	Reduced intramolecular pegRNA loops	NT	Nelson et al. (2022)
	PBS reduction	Shorter PBS (7-8 bp)	Reduced intramolecular pegRNA autoinhibition	OS	Liu et al. (2021), Ponnienselvan et al. (2023)
	PBS Tm	Tm of PBS is approximately 30 °C	Increased prime editing	OS	Lin et al. (2021)
	Composite promoter	Composite promoter (RNApolII/RNApolIII)	Particularly increased level of sgRNA transcription	OS, TA, ZM	Li et al. (2022a), Ni et al. (2023), Qiao et al. (2023), Vu et al. (2024)
	spegRNA	Introducing single-sense synonymous mutations in first bases after nicking site (either +1, +2/5, +3/+6)	Inhibition of the MMR system	OS	Li et al. (2022c), Xu et al. (2022b)
	apegRNA	Substituting the G/A pair at the hairpin 1 base with a C/G	Increased pegRNA stability	NT	Li et al. (2022c)
PE specificity	nCAS9 N854A	N854A mutation	Suppressing residual DSB generation activity of nickase	NT	Lee et al. (2023)

NT, Not Tested in plants; TA, Triticum aestivum; OS, Oryza sativa; ZM, Zea mays.

### Cas9 and reverse transcriptase mutations and modifications improve the efficiency and/or specificity of PE

#### nCas9 modifications

One strategy to improve PE is to introduce specific mutations into SpCas9 that enhance its editing efficiency. Simultaneous introduction of the R221K and N394K mutations have been shown to double editing efficiency, across eight distinct human genomic targets (Spencer and Zhang, 2017). Located at the interface of the REC1 and REC2 domains, these mutations probably facilitate HNH positioning and SpCas9 cleavage activity. Incorporating these mutations into the prime editor, led to a fourfold increase in editing efficiency in plants systems (Li J. et al., 2022; Ni et al., 2023; Qiao et al., 2023). These mutations may affects nickase’s cleavage efficiency and/or enhance pegRNA binding to its target. Unlike the D10A nickase, the H840A nickase which cut the nontargeted DNA strand has residual DSB activity (Lee et al., 2023). This residual activity is responsible for the significant rate of mutations induced by the NHEJ repair system. The mutation rate depends on the region targeted (Lee et al., 2023). Introduction of N854A and N863A mutations into the H840A nickase eliminate its residual DSB activity while maintaining editing efficiency, thus significantly enhancing specificity (Lee et al., 2023). This modification is obviously of interest for improving the specificity of PE for use in gene therapy but would also be useful in plants and use D10A nickase in prime editors thus represent also an interesting alternative to reduce indel formation but to our knowledge, it has never been tested in plants.

#### Reverse transcriptase modifications

The RNAse H domain of MMLV degrades viral RNA in heteroduplexes after retrotranscription. Its deletion have been shown to stabilize the pegRNA/DNA heteroduplex during PE, leading to a threefold increase editing efficiency as demonstrated in (Zong et al., 2022; Ni et al., 2023). Removing of both the RNAse H and the adjacent connection domain from MMLV reverse transcriptase completely abolished PE, suggesting the connection domain is necessary for the proper function of MMLV (Ni et al., 2023). Furthermore, the same authors demonstrated that the incorporation of a nucleocapsid protein, functioning as a chaperone for MMLV reverse transcriptase, also improved PE efficiency. Additional studies suggest that while the RNAse H-deficient version generally improves prime editing efficiency, it may lead to increased indel mutations with highly structured reverse transcriptase templates (RTT) (Doman et al., 2023). To improve pegRNA reverse transcription, researchers analyzed the effects of specific mutations described to improve MMLV reverse transcriptase activity in wheat PE (Ni et al., 2023). Introducing the V223A mutation resulted in an average sixfold increase in editing efficiency. This mutation has been associated previously with increase processivity and faster reverse transcription compared to the wild type enzyme (Paliksa et al., 2018). Other retrovirus-derived reverse transcriptases have been tested, such as those derived from cauliflower mosaic virus (CaMV) to enhance prime editing in rice and wheat (Lin et al., 2020). Although the CaMV-based prime editor works with an efficiency comparable to MMLV (Lin et al., 2020), none have been found to be superior. MMLV reverse transcriptase comes from animal systems and has been optimized for prime editing (Anzalone et al., 2019). CaMV-derived reverse transcriptase, which comes from plants, has never been optimized. We can only speculate that introducing mutations analogous to those in MMLV (e.g., D200N, L603W, T306K, W313F, and T330P) could potentially improve the efficiency of CaMV-based prime editors in plant systems (Anzalone et al., 2019). Introducing these or structurally analogous mutations, guided by predicted reverse transcriptase protein structures has been shown to significantly enhance PE efficiency of alternative RTs (Doman et al., 2023). Despite extensive efforts, involving mutagenesis and phage-assisted evolution, none of the reverse transcriptases developed have surpassed MMLV’s efficiency in human cells prime editing applications (Liu et al., 2022; Doman et al., 2023). Interestingly, Cao et al. (2024) reported that PE6c, incorporating an engineered RT from the yeast Tf1 retrotransposon, and PE6d, a MMLV variant, achieved 2–3.5-fold higher editing efficiency compared to PE3. Conversely, Xu et al. (2024) reported that PE6c was less efficient and PE6d equivalent to PE2 for small edits insertion in rice. Further experiments are thus needed to provide a final conclusion on the efficiency of these new versions of PE in plants. These novel, more compact RTs with efficiencies similar to MMLV, are particularly promising for applications where vector size is a limiting factor, such as RNA virus-mediated delivery systems for plant prime editing.

### PegRNA improvements

#### PegRNA structure, folding, stability and expression

PegRNA secondary structure can lead to misfolding, which negatively impacts editing efficiency by promoting unfavorable intramolecular interactions. Although sgRNA are less prone to misfolding, their secondary structures can still influence editing. For example, in a study testing the structural determinants of the editing efficiency of many sgRNAs, the self-folding energy and Tm of the sgRNA were among the factors that were found to most strongly influence editing (Wang et al., 2019). Key factors influencing pegRNA functionality include: its availability and stability, the resistance of its 3′-end to exonucleases degradation, and its secondary structure, which affects interactions with the nCas9-MMLV complex and the hybridization efficiency of the PBS to the non-targeted strand. While sgRNAs are largely shielded from 3′exonuclease activity upon binding to SpCas9, the addition of 3′extensions in pegRNAs exposes them to degradation. This degradation results in formation of competing truncated pegRNAs that can still associate with nCas9 but are ineffective for prime editing (Nelson et al., 2022). Incorporating structured RNA motifs, such as the 42-nucleotides evopreQ1, at the of 3′-end of pegRNA enhances their stability and has led to significant improvements in PE efficiency (Nelson et al., 2022) in human cells. These improved pegRNA were termed enhanced pegRNA (epegRNA). To minimize unintended interactions between the structured motif and the pegRNA, the authors suggest inserting an 8-nucleotide random linker designed using the pegLIT software. Finally, they also demonstrated that incorporation of evopreQ1 could influence pegRNA transcription leading to a recommendation for using enhanced promoters to avoid a trade-off between pegRNA protection and transcription (Nelson et al., 2022). Additional 3′modifications to pegRNAs have been shown to improved PE efficiency (Liu et al., 2021; Li et al., 2022b), probably by providing increase resistance to exonuclease degradation. Implementing the evopreQ1 motif at the 3′ end of pegRNA, *i.e*., using epegRNA, significantly improved the PE efficiency in several plant species (Jiang et al., 2022; Li J. et al., 2022; Zong et al., 2022; Ni et al., 2023; Qiao et al., 2023).

An intrinsic feature of pegRNA design is the potential for intramolecular base pairing between the primer binding site (PBS) and the spacer sequence, as both target overlapping regions. This intramolecular interaction is responsible for an autoinhibitory effect that affects target binding and initiation of reverse transcription (Liu et al., 2021; Ponnienselvan et al., 2023). This autoinhibitory effect was demonstrated by substituting PE with Cas9 and using pegRNA to cleave the target, revealing reduced activity (Liu et al., 2021; Vu et al., 2022; Ponnienselvan et al., 2023). Using pegRNA instead of sgRNA has been shown to abolish or diminish editing efficiency in both mammalian and plant cells (Ponnienselvan et al., 2023). Historically, the most efficient PBSs were 11–13 nucleotides long (Anzalone et al., 2019; Kim et al., 2021), while recent studies found that shortening the PBS to 7–8 nucleotides, alleviated autoinhibition, restoring editing with Cas9 and pegRNA (Liu et al., 2021; Ponnienselvan et al., 2023).

How can the conflicting findings regarding optimal PBS lengths be reconciled? Historically, pegRNAs lacked 3′protected structures probably leading to partial degradation by endogenous exonucleases necessitating longer PBS regions to maintain functionality. Integration of 3′protectives structures makes it possible to use shorter PBSs and limiting or even eliminating the autoinhibitory effect associated with longer PBSs in mammalian cells (Liu et al., 2021; Ponnienselvan et al., 2023). In plants, the impact remains unclear, since reducing the PBS size did not enhance editing efficiency in tomato (Ponnienselvan et al., 2023). However, the pegRNAs used in these experiments were not 3′-protected and rendering them vulnerable to partial degradation by exonucleases, as noted by authors. The melting temperature of the PBS is another critical parameter influencing prime editing efficiency. The optimal melting temperature of PBS for a large set of pegRNAs in rice and mammalian cells corresponded to optimal growth temperatures of the host cells: 30 °C for rice (Lin et al., 2021) and 37 °C for mammalian cells (Ponnienselvan et al., 2023), respectively.

Another strategy to improve PE for indels is to modify the hairpin 1 of the pegRNA. Unlike shorter sgRNA, pegRNA carry additional RTT and PBS sequences at their 3′end, which can disrupt hairpin 1 stability and lead to overall pegRNA misfolding. Replacing the G/A pair at the base of hairpin 1 with a C/G pair yielded the so-called ‘apegRNA,’ enhancing prime editing efficiency roughly threefold (Li et al., 2022c). Interestingly, this approach echoes the GOLD strategy, developed to improve genome editing by increasing stability of hairpin 1 (Riesenberg et al., 2022). Authors have shown that suboptimal sgRNA suffer from unwanted 3′-spacer base pairing with the spacer and locking hairpin 1 restored their activity (Riesenberg et al., 2022). GOLD stabilization strategy to pegRNA may similarly enhance prime editing efficiency and warrants experimental evaluation. To the best of our knowledge, these simple yet elegant strategies have not yet been evaluated in plants.

Multiple studies have reported that employing composite promoters (Li J. et al., 2022; Ni et al., 2023; Qiao et al., 2023) or utilizing viral amplicons (Vu et al., 2024) can substantially enhance prime editing efficiency in plants. There are many reasons for this: increased pegRNA transcription levels, as RNA polymerase II promoters are more effective at transcribing long RNAs; and improved pegRNA folding facilitated by incorporating a 5′tRNA and a 3′HDV ribozyme, which are cleaved during maturation. This is particularly true for low-efficiency sgRNAs, indicating that higher expression levels of pegRNA/sgRNA are necessary to achieve effective edition (Yuen et al., 2017).

#### Manipulation of the repair pathway

The mismatch repair (MMR) system corrects errors during replication by detecting mismatches and identifying the newly synthesized strand through nearby DNA nicks. Anzalone et al. introduced the PE3 system, which employs an additional sgRNA to nick the unedited strand at a distance from the edited strand, thereby enhancing the likelihood that the MMR system will replace it using the edited strand as a template (Anzalone et al., 2019). However, simultaneous nicking of both DNA strands can lead to DSBs, which may be repaired by the non-homologous end joining (NHEJ) pathway, potentially resulting in indel mutations. To mitigate this issue, Anzalone et al. developed the PE3b system, where the additional sgRNA targets the edited strand, enabling for sequential nicking limiting the incidence of unintended indel (Anzalone et al., 2019). Unfortunately, the PE3 and PE3b versions did not improve PE in plants for unknown reasons (Lin et al., 2020; Xu R. et al., 2020).

The 3′ ssDNA flap carrying the edited sequence competes with the 5′flap derived from the unedited strand for integration into the genome (Anzalone et al., 2019). Two approaches have therefore been explored to enhance 3′flap incorporation during prime editing. The first approach is to inhibit repair pathways that are deleterious for PE and 3′flap degradation. In bacteria, deletion of three exonucleases has been shown to enhance prime editing efficiency by up to 100-fold (Zhang et al., 2024). Combining PE with Cas12a-mediated CRISPR interference of exonucleases further boosts editing efficiency in bacterial models (Zhang et al., 2024). In mammalian cells, suppression of specific MMR components has also been found to increases prime editing efficiency (Chen P. J. et al., 2021; Ferreira da Silva et al., 2022). Unfortunately, attempts to replicate MMR inhibition strategies in plants, such as coexpressing dominant-negative forms of MLH1 dh and MutS, have not significantly improved PE in rice and tomato (Li J. et al., 2022; Vu et al., 2022). However, RNAi-mediated knockdown of OsMLH1 improved PE in rice (Liu X. et al., 2024). While the precise genetic factors influencing PE in plants remain unclear, comparisons across species suggest that the repair pathways involved may differ significantly between bacteria, humans, and plants. In any case, of MMR inhibition must be temporary as prolonged suppression can elevate mutation rate and compromise genomic stability.

An alternative strategy involves facilitating the removal of the 5′DNA flap by incorporating a 5′to 3′exonuclease into the prime editing system. In human cells, the recruitment of bacteriophage T5 exonuclease through PP7 (*Pseudomonas* bacteriophage) RNA aptamers inserted at the pegRNA’s tetraloop has proven to be an effective system in human cells (Truong et al., 2024). This approach generally increases PE efficiency in comparison with the PEmax system, but above all, it improves PE specificity for insertion ranging from 30 to 60 bp pairs, which is consistent with the idea that this system favors integration of longer 3′flaps. A comparable strategy was applied in rice, where fusing the same to the N-terminus of the prime editor led to a 1.7- to 2.9-fold increase in editing efficiency varying with the target site (Liang et al., 2023). Interestingly, using a similar aptamer mediated exonuclease strategy as described in (Truong et al., 2024), resulted in reduced prime editing efficiency in his context (Liang et al., 2023). The authors utilized MS2 aptamers inserted in the 3′end of the pegRNA, which may have negative effect on pegRNA binding to its target compared to tetraloop insertions (Truong et al., 2024), thereby reducing PE efficiency (Liang et al., 2023).

Prime editing efficiency depends on favoring the integration of the edited DNA strand over the original wild-type strand. The Mismatch repair (MMR) system typically targets edited strand for correction, as it preferentially recognizes nicks introduced during editing. Enhancing the incorporation of the edited strand, requires to bias the MMR to favor its integration. A strategy involves inhibiting MMR by introducing multiple synonymous mutations in and around the protospacer adjacent motif (PAM). Altering the PAM or the adjacent seed region through mutations prevent nCas9 from re-binding and re-cutting the edited strand, thereby reducing MMR recognition and enhancing prime editing efficiency (Anzalone et al., 2019). Introducing multiple synonymous mutations can hinder MMR recognition, since MMR complex such as Msh2-Msh6 primarily detect single-base mismatches and small indels, whereas Msh2-Msh3 targets larger indels which is consistent with the idea that inhibiting of the MMR enhance prime editing efficiency (Ferreira da Silva et al., 2022). Consequently, multiple substitutions are less efficiently recognized by MMR, reducing the likelihood of the edited strand being corrected back to the wild-type sequence. Finally, targeting the coding strand (non-RNApolymerase template strand) of actively transcribed regions for editing may be advantageous as it is not used as a template for correction during transcription. Indeed, transcription-coupled repair, along with mismatch repair, preferentially monitors and corrects the template (non-coding) strand when heteroduplexes are present (Georgakopoulos-Soares et al., 2020). Therefore, editing the coding strand can also therefore theoretically increase the rate of prime editing, particularly when combined with introduction of multiple synonymous mutations.

This strategy of introducing multiple synonymous mutations has been effectively applied in both animal and plant cells. For instance, introducing same-sense mutations (SSMs) or silent mutations at positions +1, 5, 6, 2/5, and 3/6, relative to the nicking site, has been shown to strongly enhance PE efficiency, by an average of 350-fold, particularly for pegRNAs with very low initial efficiency (Li et al., 2022c). These modified pegRNAs, termed spegRNAs are compatible with PE2 or PE3 systems, albeit demonstrating enhanced synergistic effects when used with PE3 (Li et al., 2022c). In rice, Xu, et al. demonstrated that introducing mutations within the RTT, at the PAM or PAM-proximal region strongly enhanced prime editing efficiency (Xu W. et al., 2022). Li, X. et al. proposed guidelines for introducing SSMs in single-base PE, suggesting placement at +3/+6 when substituting the first base after nicking, at +1 for the second base, and at +2/+5 the third base (Li et al., 2022c). This strategy maintains the reading frame and ensures that only the targeted amino acid is modified. Combining spegRNA and apegRNA, where apegRNA correspond to a substitution of the G/A pair at the hairpin 1 base by a C/G, have shown synergistic effects (Li et al., 2022c). Interestingly, sapegRNAs also enhance PE efficiency in the PE2 system by approximately threefold, although this improvement is less pronounced compared to their effect in the PE3 system (Li et al., 2022c).

#### Dual pegRNA for prime edition

An interesting approach to enhance prime editing efficiency is to use a dual pegRNA system, where two pegRNA are designed to introduce identical modifications on both the 5′and 3′strands [see, for example, (Lin et al., 2021; Choi et al., 2022)]. This strategy therefore requires the design of two pegRNAs along with compatible nicking sites to facilitate simultaneous editing on both strands. Tools like PlantPegDesigner (Lin et al., 2021) provide design assistance for dual pegRNA strategies when applicable, aiming to optimize PE efficiency. This strategy is interesting, although its applicability depends on the availability of suitable target sites.

### Insertion by dual pegRNA prime editing

Prime editing allows for the insertion of short DNA sequences using a single pegRNA. Anzalone et al. demonstrated this by integrating sequences such as a His6 tag (18 bp), a FLAG epitope tag (24 bp), or an extended loxP site (44 bp) into the HEK3 locus using the PE3 version, achieving efficiencies ranging approximately from 60% to 20% (Anzalone et al., 2019). In plant systems, initial triasl showed low insertion efficiencies that declined sharply with increasing insert size: from 3% for a 3 bp insertion, dropping to 0.3% for 15 bp and becoming undetectable for insertions exceeding 15 bp (Lin et al., 2020).

**FIGURE 4:**
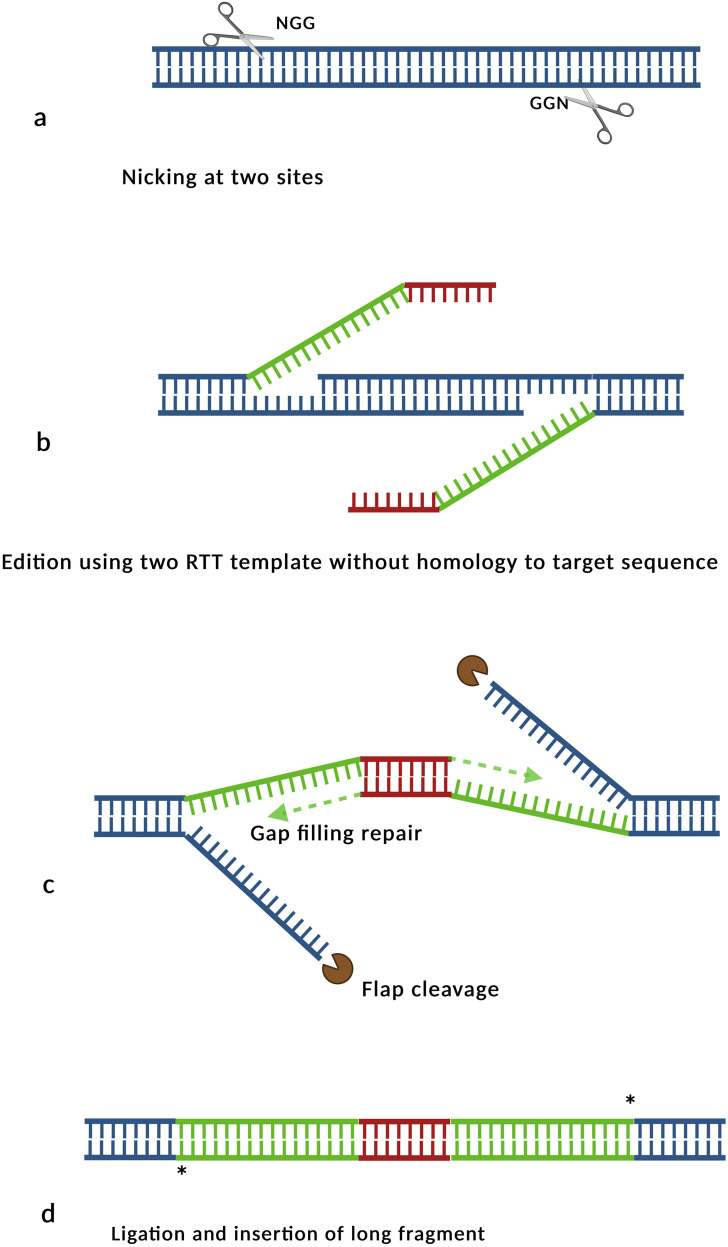
Schematic representation of insertion via GRAND. **(a)** Following nicking and PBS annealing, **(b)** the RT templates are extended by reverse transcriptase, generating DNA flaps composed of specific (green) and complementary (red) sequences. **(c)** The 5′flaps are processed, and the overlapping regions are resolved through gap filling, **(d)** the original genomic fragment is excised and replaced by the newly synthetized DNA segment. Inspired by Wang J. et al. (2022).

The use of optimized dual pegRNAs has markedly improved insertion efficiencies in plants. For example, the integration of a 36 bp Lox66 sequence achieved an average insertion rate of 25% across eight distinct targets with efficiencies reaching up 50% (Sun et al., 2024). The GRAND (Genome-wide Rapid and Accurate DNA insertion) strategy (Figure 4), utilizing dual pegRNAs, facilitated the insertion of 150 bp and 250 bp fragments with efficiencies of 60% and 30%, respectively. However, insertion efficiencies declined significantly for fragments exceeding 400 bp (Wang J. et al., 2022). The GRAND approach employs two RTTs that are partially complementary to each other, ensuring no sequence homology with the targeted genomic region, thereby minimizing unintended recombination events (Figure 4). Key factors for the success of this dual peg technology include designing the RTTs devoid of microhomology with the target sites and insuring that complementarity between the RTTs is restricted to their terminal regions (Wang J. et al., 2022).

Comparable dual peg methodologies have been used by other research groups (see, for example, (Anzalone et al., 2022)), confirming the efficiency of this knock-in strategy in plants (Li et al., 2023; Sun et al., 2024; Zhong et al., 2024), with insertion rates exceeding 20%–30%. Interestingly, deletion efficiencies are also greater in plants utilizing dual pegs compared to those employing PE3s (Liu M. et al., 2024). Moreover, the introduction of multiple synonymous base mutations within the annealing regions RTT templates significantly enhanced insertion rate and, more importantly, enabled the generation of homozygous insertions in primary transformants via the PrimeDel approach. In rice, the PE6d variant has demonstrated superior performance over PE2 for small tag insertions (Xu et al., 2024). This variant combines the deletion of the RNAse H domain of MMLV along specific mutations (T128N/N200C/V223Y) to enhance reverse transcription processivity (Doman et al., 2023; Xu et al., 2024).

Finally, one of the limitations of dual pegRNAs strategies is related to the transcriptional capacity of U3 and U6 promoters, which are constrained in their ability to transcribe extended extended RTT sequences. In studies using dual pegRNA approaches to induce deletions (primeDel), the authors reported that stable genomic integration of prime editing constructs led to a progressive increased in deletion frequencies over time, surpassing those achieved through transient expression (Choi et al., 2022). These findings underscore the importance of using composite promoters, which not only enhance pegRNA transcription levels but also help the efficient transcription of RTTs exceeding 300 bp, as the U6 and U3 promoters are unable to efficiently produce transcripts larger than 300 bp. This limitation likely contributes to the observed decline in insertion efficiency of the GRAND technology when targeting sequences exceeding 400 base pairs (Wang J. et al., 2022).

Template-jumping PE (TJ-PE) is an alternative prime editing strategy inspired by the natural insertion mechanisms of retrotransposons (Zheng et al., 2023). This approach utilizes a single TJ-pegRNA that contains the desired insertion sequence flanked by two primer binding sites (PBSs). Following the initial retrotranscription, initiated at the 3′nicked end, a second sgRNA induces a nick on the opposite DNA strand. This newly exposed 3′ end, complementary to the second PBS, serves as a primer for reverse transcription of the opposite strand. Insertion efficiencies achieves with TJ-PE are approximatively, 50%, 35% and 10% for 200, 300 and 500 bp fragments, respectively, and for larger fragments such as 800 bp, the efficiency drops to around 2%. These efficiencies are comparable to those achieved with GRAND technology; however, TJ-PE technology offers a theoretically simpler approach, requiring only a single pegRNA and sgRNA. This technology has not yet been tested in plants.

### Dual pegs and site-specific integrases for the insertion of long sequences

To address the limitations associated with inserting large DNA, recent strategies have been combined dual prime editing techniques with site-specific integrases, facilitating recombination-based integration of extended DNA fragments. Anzalone et al. (2022) used their twinPE approach, utilizing dual pegRNA, to insert a homozygous attB sequence at the CCR5 locus. Subsequent transfection with a codon-optimized Bxb1 serine integrase and a donor DNA in plasmid flanked 5′by an attP site resulted in knock-in efficiencies ranging from 12% to 17% for a 5.6 kb sequence. Yarnall et al. (2023) adopted a comparable methodology, demonstrating that Cre/Lox systems were less effective than serine integrase for integrating long DNA sequences. Among the serine integrase tested, Bxb1 exhibited superior performance, achieving a 15% insertion rate for a 900 bp fragment. This initial system, termed PASTEv1, featured a fusion of the Bxb1 serine integrase with the MMLV. Subsequent optimizations, including modifications to the linker region, the MMLV, Bxb1 sequences lead to the development of PASTEV2, which achieved an enhancer insertion efficiency of 30% (Yarnall et al., 2023). Integration of PASTEv2 with an optimized version of pegRNA (atgRNAv2), culminated in PASTEV3, which facilitated the insertion of DNA fragments up to 36 kb at two distinct genomic loci. To streamline the design of optimized pegRNAs, the authors developed predictive software for atgRNA construction. Furtheremore, by using various serine integrases and attB/attP dinucleotide pairings, they demonstrated the feasibility of multiplexing the atgRNA strategy (Yarnall et al., 2023).

A similar strategy was implemented for targeted insertion of large fragments in rice (Sun et al., 2024). Initially, the authors optimized dual pegRNA-mediated prime editing, achieving average insertion efficiency of 25% across eight distinct targets in protoplasts and up to 40% in regenerated plants. Sun et al. reported that the use of the RNA polymerase II promoter is two times more effective than the use of RNA polymerase III promoter to generate large dual pegRNA insertions. Notably, use of RNA pol II promoter is still efficient for larger inserts, albeit with moderate reported rates of approximately 8% for 400 bp, 3% for 500 bp and below 1% for 720 bp sequences (Sun et al., 2024). The study further demonstrated that Cre and FLP recombinase systems are among the most effective for plant genome engineering. Authors inserted Lox66 or F1 m2 sequences using dual pegRNAs, followed by re-transformation of the resistant callus with constructs expressing either Cre of FLP integrase. The optimized version, termed PrimeRootV3.0, is capable of integrating sequences ranging from 1.4 kb to over 11 kb achieving insertion efficiency of approximately 3%–6% through sequential transformation methods utilizing either biolistic delivery or *A. tumefaciens*.

### Future directions: genome editing technologies for plant breeding

#### Integrating SpCas9 enhancements into present and future genome editing technologies

Improving the efficiency and specificity of base editing (BE) and prime editing (PE) requires leveraging improvements made in native SpCas9 alongside technology-specific modifications. A rational approach to SpCas9-derived editing technologies should incorporate these optimizations upstream to develop more efficient constructs. For instance, incorporating introns into the SpCas9 has been shown to significantly boost expression and editing efficiency in dicotyledonous plants (Grutzner et al., 2021), suggesting potential benefits for both BE and PE. Similarly, composite promoters have been demonstrated to enhance the expression of inefficient sgRNAs and markedly increase PE efficiency in plant systems (Li J. et al., 2022; Ni et al., 2023; Qiao et al., 2023). Composite promoters should be particularly important for long RNA transcription and multiplexing strategies, which will be crucial for breeding applications. Further, increasing SpCas9 editing efficiency directly enhances PE efficiency (Li J. et al., 2022; Ni et al., 2023; Qiao et al., 2023), as demonstrated in PEmax, which incorporates double mutations in nCas9 (Spencer and Zhang, 2017) or a bipartite nuclear localization signal (BP-NLS) to improve nuclear targeting and editing rates compared to single NLSs (Develtere et al., 2024). The development of SpCas9-derived technologies can therefore benefit from advances in SpCas9’s efficacy and specificity, and must therefore be taken into account in any current or future editing technology. GE and PE technologies have proven valuable in plant breeding, contributing to traits such as enhanced grain quality (Zhou et al., 2019) and broad-spectrum resistance to bacterial blast (Gupta et al., 2023) in rice. Notably, genome-edited crops like GABA-enriched tomatoes (Waltz, 2022) and high-oleic acid soybean (Demorest et al., 2016) have reached commercial markets. Beyond efficiency, specificity will become a critical factor for the routine application of genome editing technologies in breeding programs, particularly within Europe. The European Commission has proposed a threshold of 20 genetic modifications, mirroring changes achievable through conventional breeding, to classify such genome-edited plants equivalently to traditional bred counterparts (Organisms et al., 2024). This includes the targeted insertion of a contiguous DNA sequence already present within the gene pool.

#### Mastering DNA repair pathways: a key to efficient genome editing

Genome editing technologies rely on endogenous DNA repair mechanisms, with numerous modifications designed to modulating specific pathways to increase editing efficiency and precision. For example, MMEJ can be exploited to induce targeted deletions by carefully selecting sgRNA that promote this repair pathway (Martinez-Galvez et al., 2021). A novel Cas9 variant, vCas9, introduces staggered DNA cuts, thereby favoring repair via MMEJ and HDR pathways over NHEJ (Chauhan et al., 2023). Base editing (BE) techniques modulates DNA repair by inhibiting BER through the use uracil DNA glycosylase inhibitors (UGIs). Enhanced versions like BE4 and BE4max employ dual UGI system to strengthen this inhibition, while nicking the unedited DNA strand stimulate MMR to favor insertion of the desired edit (Komor et al., 2017; Koblan et al., 2018). Prime Editing (PE) efficiency can be enhanced by suppressing of MMR, either through conditional RNAi targeting OsMLH1 in rice or by introduction of multiple silent mutations (SSMs) within the RTT of pegRNA (Xu W. et al., 2022). PE3b enhances editing efficiency by introducing a second sgRNA to nick the unedited strand, facilitating precise repair stimulating MMR of the unedited strand (Anzalone et al., 2019). Beyond direct genome editing, CRISPR-based transcriptional modulation techniques such as CRISPR activation (CRISPRa) and interference (CRISPRi) can be also use to targeting key repair genes using catalytically inactive sgRNA termed dead sgRNAs (dsgRNAs) (Dahlman et al., 2015; Ye et al., 2018), a promising avenue to enhance efficiency and specificity of PE, BE and emerging editing technologies. Furthermore, variations in DNA repair pathway regulation across species may account for observed differences in editing efficiencies, especially between monocotyledonous and dicotyledonous plants. This is not new, the efficiency of stable T-DNA transformation in plants has long been associated with the activity of NHEJ and MMEJ repair pathways (Qi et al., 2013; Saika et al., 2014). Overexpression of Ku80, a pivotal protein in the NHEJ pathway, has been shown to improve transgene integration (Li et al., 2005). A deeper understanding of DNA repair mechanisms and their interplay with genome editing technologies is crucial to overcome existing limitations for applications of plant genome editing technologies.

#### Genome editing for crop improvement: overcoming transformation barriers

One of the main limitations to the use of genome editing technologies in plants is genetic transformation, which is still restricted to certain species and/or genotypes. For a recent review on this topic, see (Wang et al., 2025). Among transformation approaches, protoplast-based and biolistic methods offer the advantage of generating transgene-free edits, but they remain limited by laborious regeneration protocols and genotype dependency in most species. Biolistic delivery enables transformation of a wide range of tissues but the approach often causes extensive DNA rearrangements. By contrast, *A. tumefaciens* mediated transformation remains the most widely used and reliable system for stable integration with fewer somaclonal variants, although many agronomically important species remain recalcitrant and genotype dependent, underscoring the need to expand its applicability to diverse tissues and resistant genotypes.

To overcome this, strategies have been developed to improve transformation efficiency by modulating dedifferentiation regulators (such as GRF, WUSCHEL (WUS) and BABY BBOL (BBM) (Lowe et al., 2016; Debernardi et al., 2020). However, the constitutive expression of these pivotal developmental genes can lead to adverse effects, highlighting the need of alternative methods to enhance transformation and regeneration. An alternative promising strategy involved CRISPR activation (CRISPRa) using dead single-guides RNA (dsgRNAs) with inducible promoters enabling the temporal activation of these regulators within specific time frames (Dahlman et al., 2015). Furthermore, the same strategy can be used to facilitate stable insertions by enhancing the activity of the non-homologous end joining (NHEJ) repair pathway (Li et al., 2005).

In vegetatively propagated crops, the inability to eliminate transgenes through segregation necessitates the development of transgene-free genome editing approaches. This challenge is mainly being addressed by transient transformation of protoplasts, which avoids stable integration of transgenes while still allowing precise genetic modification (Gu et al., 2021). Another approach involves grafting wild-type shoots onto transgenic donor rootstocks that express mobile RNA versions of SpCas9 and sgRNA (Yang et al., 2023). However, efficient regeneration remains a significant hurdle for many species; the use of dedifferentiation regulators has been shown to enhance this process (Lowe et al., 2016; Debernardi et al., 2020). Moreover, the editing efficiency achieved trhough grafting strategies remains too low, often below 0.1%, rendering them impractical for routine breeding applications (Yang et al., 2023). Ultimately, for genome editing to be routinely applied in the breeding of vegetatively propagated crops, it is essential to optimize both transformation and editing efficiency. Without selection markers, the frequency of regenerated plants harboring the desired edit depends on both the success of transformation success and the efficiency of the editing process. Enhancing both transformation and editing efficiencies will minimize the screening required to identify desired mutations, thereby accelerating the adoption of genome editing in crop improvement.

#### Extending the reach of genome editing: identifying and introducing agronomic alleles

A key challenge in crop improvement is identifying alleles of agronomic interest and tailoring genome editing strategies accordingly. The majority of beneficial alleles have been discovered in model species, limiting their direct application to a wide range of crops species. Addressing this limitation, requires a comprehensive catalogue of beneficial alleles and the identification of their orthologs in target crop species through translational biology, thereby expending the repertoire of potential genetic improvements (Inze and Nelissen, 2022). The specific nature of the desired genetic alteration dictates the choice of genome editing technology.

For example, CRISPR/Cas9 can efficiently generate simple gene knockouts, exemplified by the yield-enhancing GS2 alleles in rice (Wang W. et al., 2022). However, intricate genetic modifications require advanced technologies: for example, base editing (BE) facilitates the introduction of the NRT1.1B allele to enhance nitrogen use efficiency (NUE) (Lu and Zhu, 2017), whereas prime editing (PE) enables the precise insertion of heat stress-responsive elements (HSEs) into invertase promoters in rice and tomato to maintain yield at high temperatures (Lou et al., 2025).

Addressing structural variations, including presence-absence variants (PAVs), like the *SUB1* (Xu et al., 2006) or *PSTOL1* (Gamuyao et al., 2012) genes in rice, remain challenging; however, emerging PE-based technologies capable of integrating kilobase-scale DNA sequences offer promising solutions (Sun et al., 2024). Combining the effects of these favorable alleles across various target species and genetic backgrounds is a crucial step. In rice, introduction yield-enhancing mutations across various genotypes has resulted in variable outcomes, likely due to unpredictable complex epistatic interactions (Shen et al., 2018). These findings highlight the need for comprehensive field trials and the continuous refinement of genome editing strategies.

This review offers an overview of recent progress in plant genome editing, from SpCas9 to prime editing. Genome editing technologies are becoming increasingly efficient and precise, thereby accelerating plant breeding and facilitating functional gene analysis. Among the most significant advances, multiplexing is pivotal to expediting breeding programs, as it allows the concurrent introduction of multiple advantageous mutations (Zhou et al., 2019; Zhou et al., 2024). Moreover, due to its versatility in facilitating both simple and complex genetic modifications, prime editing holds promise for large-scale, multi-target genome alterations, potentially revolutionizing crop improvement strategies (Gupta et al., 2024). Nevertheless, important limitations remain: the difficulty of achieving efficient transformation in many agronomically relevant species, restrictions on knock-in size, and heterogeneous prime editing efficiencies across targets and species. Future efforts should focus on improving transformation efficiency and delivery systems, as well as developing more robust and efficient prime editors for plant breeding and functional genomics. Finally, we have not yet reached the end of the golden path of genome editing with the advent of new emerging technologies like the bridge RNA (Durrant et al., 2024).

## Glossary

**CRISPR**: clustered regularly interspaced short palindromic repeats
**Cas**: Cas-associated protein
**Cas9**: Cas-associated protein 9
**crRNA**: CRISPR RNAs
**nCas9**: nickase Cas9 (either D10A or H840A)
**dCas9**: dead Cas9 (D10A and H840A)
**SpCas9**: *Streptococcus pyogenes* Cas9
**SaCas9**: *Staphylococcus aureus* Cas9
**crRNA**: CRISPR RNA
**tracrRNA**: transactivating CRISPR RNA
**sgRNA**: single guide RNA
**hpsgRNA**: hairpin sgRNA
**dsgRNA**: dead sgRNA
**pegRNA**: prime editing guide RNA
**scRNA**: scaffold RNA
**PAM**: protospacer adjacent motif
**REC1, REC2, REC3**: recognition domain, 1, 2 and 3
**BH**: bridge helix
**HNH**: domain that cleaves the DNA strand complementary to the spacer
**RuvC**: nuclease domain that cleaves the DNA strand noncomplementary to the spacer
**Pi**: PAM interaction domain
**NUC**: nuclease domain
**TOPO**: topoisomerase domain
**CRISPRi**: CRISPR interference
CTD: C-terminal domain of SpCas9
**RNP**: ribonucleoprotein
**GE**: genome editing
**PE**: prime editing
**BE**: base editing
**rAPOBEC1**: rat cytidine deaminase
**UDG**: Uracil DNA glycosylase
**BER**: base excision repair
**UGI**: uracil glycosylase inhibitor
**CBE**: cytosine base editor
**ABE**: adenine base editor
**CGBE**: C-to-G base editor
**DBE**: dual base editor
**AI**: artificial intelligence
**cNHEJ**: classical nonhomologous end joining
**MMR**: mismatch repair
**HDR**: homologous DNA repair
**Csy4**: CRISPR/Cas9 subtype Ypest protein 4
**TDR-HDR**: Tandem repeat HDR
**HH**: hammerhead ribozyme
**HDV**: hepatitis delta virus ribozyme
**RT**: reverse transcriptase
**MMLV**: Moloney murine leukemia virus
**PBS**: primer binding site
**RTT**: reverse transcriptase template
**GRAND**: Genome-wide Rapid and Accurate DNA insertion
**SSB**: single-strand break
**DSB**: double-strand break
**dsDNA**: double-stranded DNA
**ssDNA**: single-stranded DNA
**CaMV**: cauliflower mosaic virus
**SSM**: same-sense mutation

## References

[B1] Aird E. J. Lovendahl K. N. St Martin A. Harris R. S. Gordon W. R. (2018). Increasing Cas9-mediated homology-directed repair efficiency through covalent tethering of DNA repair template. Commun. Biol. 1, 54. 10.1038/s42003-018-0054-2 30271937 PMC6123678

[B2] Ali Z. Shami A. Sedeek K. Kamel R. Alhabsi A. Tehseen M. (2020). Fusion of the Cas9 endonuclease and the VirD2 relaxase facilitates homology-directed repair for precise genome engineering in rice. Commun. Biol. 3, 44. 10.1038/s42003-020-0768-9 31974493 PMC6978410

[B3] Alok A. Sandhya D. Jogam P. Rodrigues V. Bhati K. K. Sharma H. (2020). The rise of the CRISPR/Cpf1 system for efficient genome editing in plants. Front. Plant Sci. 11, 264. 10.3389/fpls.2020.00264 32296449 PMC7136500

[B4] Anders C. Niewoehner O. Duerst A. Jinek M. (2014). Structural basis of PAM-dependent target DNA recognition by the Cas9 endonuclease. Nature 513, 569–573. 10.1038/nature13579 25079318 PMC4176945

[B5] Anzalone A. V. Randolph P. B. Davis J. R. Sousa A. A. Koblan L. W. Levy J. M. (2019). Search-and-replace genome editing without double-strand breaks or donor DNA. Nature 576, 149–157. 10.1038/s41586-019-1711-4 31634902 PMC6907074

[B6] Anzalone A. V. Gao X. D. Podracky C. J. Nelson A. T. Koblan L. W. Raguram A. (2022). Programmable deletion, replacement, integration and inversion of large DNA sequences with twin prime editing. Nat. Biotechnol. 40, 731–740. 10.1038/s41587-021-01133-w 34887556 PMC9117393

[B7] Babu K. Amrani N. Jiang W. Yogesha S. D. Nguyen R. Qin P. Z. (2019). Bridge helix of Cas9 modulates target DNA cleavage and mismatch tolerance. Biochemistry 58, 1905–1917. 10.1021/acs.biochem.8b01241 30916546 PMC6496953

[B8] Babu K. Kathiresan V. Kumari P. Newsom S. Parameshwaran H. P. Chen X. (2021). Coordinated actions of Cas9 HNH and RuvC nuclease domains are regulated by the Bridge helix and the target DNA sequence. Biochemistry 60, 3783–3800. 10.1021/acs.biochem.1c00354 34757726 PMC8675354

[B9] Banakar R. Eggenberger A. L. Lee K. Wright D. A. Murugan K. Zarecor S. (2019). High-frequency random DNA insertions upon co-delivery of CRISPR-Cas9 ribonucleoprotein and selectable marker plasmid in rice. Sci. Rep. 9, 19902. 10.1038/s41598-019-55681-y 31882637 PMC6934568

[B10] Barrangou R. Fremaux C. Deveau H. Richards M. Boyaval P. Moineau S. (2007). CRISPR provides acquired resistance against viruses in prokaryotes. Science 315, 1709–1712. 10.1126/science.1138140 17379808

[B11] Bravo J. P. K. Liu M.-S. Hibshman G. N. Dangerfield T. L. Jung K. Mccool R. S. (2022). Structural basis for mismatch surveillance by CRISPR–Cas9. Nature 603, 343–347. 10.1038/s41586-022-04470-1 35236982 PMC8907077

[B12] Briner A. e. Donohoue P. d. Gomaa A. a. Selle K. Slorach E. m. Nye C. h. (2014). Guide RNA functional modules direct Cas9 activity and orthogonality. Mol. Cell 56, 333–339. 10.1016/j.molcel.2014.09.019 25373540

[B13] Cameron P. Fuller C. K. Donohoue P. D. Jones B. N. Thompson M. S. Carter M. M. (2017). Mapping the genomic landscape of CRISPR–Cas9 cleavage. Nat. Methods 14, 600–606. 10.1038/nmeth.4284 28459459

[B14] Cao Z. Sun W. Qiao D. Wang J. Li S. Liu X. (2024). PE6c greatly enhances prime editing in transgenic rice plants. J. Integr. Plant Biol. 66, 1864–1870. 10.1111/jipb.13738 38980229

[B15] Capdeville N. Schindele P. Puchta H. (2023). Increasing deletion sizes and the efficiency of CRISPR/Cas9-mediated mutagenesis by SunTag-mediated TREX1 recruitment. Plant J. 118, 277–287. 10.1111/tpj.16586 38113345

[B16] Casini A. Olivieri M. Petris G. Montagna C. Reginato G. Maule G. (2018). A highly specific SpCas9 variant is identified by *in vivo* screening in yeast. Nat. Biotechnol. 36, 265–271. 10.1038/nbt.4066 29431739 PMC6066108

[B17] Cerchione D. Loveluck K. Tillotson E. L. Harbinski F. Dasilva J. Kelley C. P. (2020). SMOOT libraries and phage-induced directed evolution of Cas9 to engineer reduced off-target activity. PLOS ONE 15, e0231716. 10.1371/journal.pone.0231716 32298334 PMC7161989

[B18] Cermak T. Curtin S. J. Gil-Humanes J. Cegan R. Kono T. J. Y. Konecna E. (2017). A multipurpose toolkit to enable advanced genome engineering in plants. Plant Cell 29, 1196–1217. 10.1105/tpc.16.00922 28522548 PMC5502448

[B19] Certo M. T. Gwiazda K. S. Kuhar R. Sather B. Curinga G. Mandt T. (2012). Coupling endonucleases with DNA end-processing enzymes to drive gene disruption. Nat. Methods 9, 973–975. 10.1038/nmeth.2177 22941364 PMC3602999

[B20] Chauhan V. P. Sharp P. A. Langer R. (2023). Altered DNA repair pathway engagement by engineered CRISPR-Cas9 nucleases. Proc. Natl. Acad. Sci. U. S. A. 120, e2300605120. 10.1073/pnas.2300605120 36881621 PMC10242711

[B21] Chen X. Rinsma M. Janssen J. M. Liu J. Maggio I. Goncalves M. A. (2016). Probing the impact of chromatin conformation on genome editing tools. Nucleic Acids Res. 44, 6482–6492. 10.1093/nar/gkw524 27280977 PMC5291272

[B22] Chen J. S. Dagdas Y. S. Kleinstiver B. P. Welch M. M. Sousa A. A. Harrington L. B. (2017). Enhanced proofreading governs CRISPR–Cas9 targeting accuracy. Nature 550, 407–410. 10.1038/nature24268 28931002 PMC5918688

[B23] Chen L. Park J. E. Paa P. Rajakumar P. D. Prekop H. T. Chew Y. T. (2021a). Programmable C:G to G:C genome editing with CRISPR-Cas9-directed base excision repair proteins. Nat. Commun. 12, 1384. 10.1038/s41467-021-21559-9 33654077 PMC7925527

[B24] Chen P. J. Hussmann J. A. Yan J. Knipping F. Ravisankar P. Chen P. F. (2021b). Enhanced prime editing systems by manipulating cellular determinants of editing outcomes. Cell 184, 5635–5652.e29. 10.1016/j.cell.2021.09.018 34653350 PMC8584034

[B25] Choi J. Chen W. Suiter C. C. Lee C. Chardon F. M. Yang W. (2022). Precise genomic deletions using paired prime editing. Nat. Biotechnol. 40, 218–226. 10.1038/s41587-021-01025-z 34650269 PMC8847327

[B26] Chylinski K. Makarova K. S. Charpentier E. Koonin E. V. (2014). Classification and evolution of type II CRISPR-cas systems. Nucleic Acids Res. 42, 6091–6105. 10.1093/nar/gku241 24728998 PMC4041416

[B27] Coelho M. A. De Braekeleer E. Firth M. Bista M. Lukasiak S. Cuomo M. E. (2020). CRISPR GUARD protects off-target sites from Cas9 nuclease activity using short guide RNAs. Nat. Commun. 11, 4132. 10.1038/s41467-020-17952-5 32807781 PMC7431537

[B28] Concordet J. P. Haeussler M. (2018). CRISPOR: intuitive guide selection for CRISPR/Cas9 genome editing experiments and screens. Nucleic Acids Res. 46, W242–W245. 10.1093/nar/gky354 29762716 PMC6030908

[B29] Daer R. M. Cutts J. P. Brafman D. A. Haynes K. A. (2017). The impact of chromatin dynamics on Cas9-Mediated genome editing in human cells. ACS Synth. Biol. 6, 428–438. 10.1021/acssynbio.5b00299 27783893 PMC5357160

[B30] Dahlman J. E. Abudayyeh O. O. Joung J. Gootenberg J. S. Zhang F. Konermann S. (2015). Orthogonal gene knockout and activation with a catalytically active Cas9 nuclease. Nat. Biotechnol. 33, 1159–1161. 10.1038/nbt.3390 26436575 PMC4747789

[B31] Dang Y. Jia G. Choi J. Ma H. Anaya E. Ye C. (2015). Optimizing sgRNA structure to improve CRISPR-Cas9 knockout efficiency. Genome Biol. 16, 280. 10.1186/s13059-015-0846-3 26671237 PMC4699467

[B32] Debernardi J. M. Tricoli D. M. Ercoli M. F. Hayta S. Ronald P. Palatnik J. F. (2020). A GRF-GIF chimeric protein improves the regeneration efficiency of transgenic plants. Nat. Biotechnol. 38, 1274–1279. 10.1038/s41587-020-0703-0 33046875 PMC7642171

[B33] Demorest Z. L. Coffman A. Baltes N. J. Stoddard T. J. Clasen B. M. Luo S. (2016). Direct stacking of sequence-specific nuclease-induced mutations to produce high oleic and low linolenic soybean oil. BMC Plant Biol. 16, 225. 10.1186/s12870-016-0906-1 27733139 PMC5062912

[B34] Develtere W. Decaestecker W. Rombaut D. Anders C. Clicque E. Vuylsteke M. (2024). Continual improvement of CRISPR-induced multiplex mutagenesis in arabidopsis. Plant J. 119, 1158–1172. 10.1111/tpj.16785 38713824

[B35] Dixit S. Kumar A. Srinivasan K. Vincent P. Ramu Krishnan N. (2023). Advancing genome editing with artificial intelligence: opportunities, challenges, and future directions. Front. Bioeng. Biotechnol. 11, 1335901. 10.3389/fbioe.2023.1335901 38260726 PMC10800897

[B36] Doench J. G. Fusi N. Sullender M. Hegde M. Vaimberg E. W. Donovan K. F. (2016). Optimized sgRNA design to maximize activity and minimize off-target effects of CRISPR-Cas9. Nat. Biotechnol. 34, 184–191. 10.1038/nbt.3437 26780180 PMC4744125

[B37] Doman J. L. Pandey S. Neugebauer M. E. An M. Davis J. R. Randolph P. B. (2023). Phage-assisted evolution and protein engineering yield compact, efficient prime editors. Cell 186, 3983–4002.e26. 10.1016/j.cell.2023.07.039 37657419 PMC10482982

[B38] Dong C. Gou Y. Lian J. (2022). SgRNA engineering for improved genome editing and expanded functional assays. Curr. Opin. Biotechnol. 75, 102697. 10.1016/j.copbio.2022.102697 35217295

[B39] Durrant M. G. Perry N. T. Pai J. J. Jangid A. R. Athukoralage J. S. Hiraizumi M. (2024). Bridge RNAs direct programmable recombination of target and donor DNA. Nature 630, 984–993. 10.1038/s41586-024-07552-4 38926615 PMC11208160

[B40] Fan T. Cheng Y. Wu Y. Liu S. Tang X. He Y. (2024). High performance TadA-8e derived cytosine and dual base editors with undetectable off-target effects in plants. Nat. Commun. 15, 5103. 10.1038/s41467-024-49473-w 38877035 PMC11178825

[B41] Ferreira Da Silva J. Oliveira G. P. Arasa-Verge E. A. Kagiou C. Moretton A. Timelthaler G. (2022). Prime editing efficiency and fidelity are enhanced in the absence of mismatch repair. Nat. Commun. 13, 760. 10.1038/s41467-022-28442-1 35140211 PMC8828784

[B42] Fu Y. Sander J. D. Reyon D. Cascio V. M. Joung J. K. (2014). Improving CRISPR-cas nuclease specificity using truncated guide RNAs. Nat. Biotechnol. 32, 279–284. 10.1038/nbt.2808 24463574 PMC3988262

[B43] Gamuyao R. Chin J. H. Pariasca-Tanaka J. Pesaresi P. Catausan S. Dalid C. (2012). The protein kinase Pstol1 from traditional rice confers tolerance of phosphorus deficiency. Nature 488, 535–539. 10.1038/nature11346 22914168

[B44] Gasiunas G. Barrangou R. Horvath P. Siksnys V. (2012). Cas9–crRNA ribonucleoprotein complex mediates specific DNA cleavage for adaptive immunity in bacteria. Proc. Natl. Acad. Sci. U. S. A. 109, E2579–E2586. 10.1073/pnas.1208507109 22949671 PMC3465414

[B45] Gaudelli N. M. Komor A. C. Rees H. A. Packer M. S. Badran A. H. Bryson D. I. (2017). Programmable base editing of A*T to G*C in genomic DNA without DNA cleavage. Nature 551, 464–471. 10.1038/nature24644 29160308 PMC5726555

[B46] Gehrke F. Schindele A. Puchta H. (2022). Nonhomologous end joining as key to CRISPR/Cas-mediated plant chromosome engineering. Plant Physiol. 188, 1769–1779. 10.1093/plphys/kiab572 34893907 PMC8968298

[B47] Georgakopoulos-Soares I. Koh G. Momen S. E. Jiricny J. Hemberg M. Nik-Zainal S. (2020). Transcription-coupled repair and mismatch repair contribute towards preserving genome integrity at mononucleotide repeat tracts. Nat. Commun. 11, 1980. 10.1038/s41467-020-15901-w 32332764 PMC7181645

[B48] Grunewald J. Zhou R. Garcia S. P. Iyer S. Lareau C. A. Aryee M. J. (2019). Transcriptome-wide off-target RNA editing induced by CRISPR-guided DNA base editors. Nature 569, 433–437. 10.1038/s41586-019-1161-z 30995674 PMC6657343

[B49] Grutzner R. Martin P. Horn C. Mortensen S. Cram E. J. Lee-Parsons C. W. T. (2021). High-efficiency genome editing in plants mediated by a Cas9 gene containing multiple introns. Plant Commun. 2, 100135. 10.1016/j.xplc.2020.100135 33898975 PMC8060730

[B50] Gu X. Liu L. Zhang H. (2021). Transgene-free genome editing in plants. Front. Genome 3, 805317. 10.3389/fgeed.2021.805317 34927134 PMC8678605

[B51] Gupta A. Liu B. Chen Q. J. Yang B. (2023). High-efficiency prime editing enables new strategies for broad-spectrum resistance to bacterial blight of rice. Plant Biotechnol. J. 21, 1454–1464. 10.1111/pbi.14049 37139586 PMC10281596

[B52] Gupta A. Liu B. Raza S. Chen Q. J. Yang B. (2024). Modularly assembled multiplex prime editors for simultaneous editing of agronomically important genes in rice. Plant Commun. 5, 100741. 10.1016/j.xplc.2023.100741 37897041 PMC10873889

[B53] Haeussler M. Schonig K. Eckert H. Eschstruth A. Mianne J. Renaud J. B. (2016). Evaluation of off-target and on-target scoring algorithms and integration into the guide RNA selection tool CRISPOR. Genome Biol. 17, 148. 10.1186/s13059-016-1012-2 27380939 PMC4934014

[B54] He R. Zhang P. Yan Y. Yu C. Jiang L. Zhu Y. (2022). Expanding the range of CRISPR/Cas9-directed genome editing in soybean. aBIOTECH 3, 89–98. 10.1007/s42994-021-00051-4 36312444 PMC9590560

[B55] Hille F. Richter H. Wong S. P. Bratovič M. Ressel S. Charpentier E. (2018). The biology of CRISPR-Cas: Backward and forward. Cell 172, 1239–1259. 10.1016/j.cell.2017.11.032 29522745

[B56] Horlbeck M. A. Witkowsky L. B. Guglielmi B. Replogle J. M. Gilbert L. A. Villalta J. E. (2016). Nucleosomes impede Cas9 access to DNA *in vivo* and *in vitro* . Elife 5, e12677. 10.7554/eLife.12677 26987018 PMC4861601

[B57] Hsu P. D. Scott D. A. Weinstein J. A. Ran F. A. Konermann S. Agarwala V. (2013). DNA targeting specificity of RNA-guided Cas9 nucleases. Nat. Biotechnol. 31, 827–832. 10.1038/nbt.2647 23873081 PMC3969858

[B58] Hu J. H. Miller S. M. Geurts M. H. Tang W. Chen L. Sun N. (2018a). Evolved Cas9 variants with broad PAM compatibility and high DNA specificity. Nature 556, 57–63. 10.1038/nature26155 29512652 PMC5951633

[B59] Hu X. Meng X. Liu Q. Li J. Wang K. (2018b). Increasing the efficiency of CRISPR-Cas9-VQR precise genome editing in rice. Plant Biotechnol. J. 16, 292–297. 10.1111/pbi.12771 28605576 PMC5785341

[B60] Hua K. Tao X. Yuan F. Wang D. Zhu J. K. (2018). Precise A.T to G.C base editing in the rice genome. Mol. Plant 11, 627–630. 10.1016/j.molp.2018.02.007 29476916

[B61] Hua K. Tao X. Han P. Wang R. Zhu J. K. (2019). Genome engineering in rice using Cas9 variants that recognize NG PAM sequences. Mol. Plant 12, 1003–1014. 10.1016/j.molp.2019.03.009 30928636

[B62] Inze D. Nelissen H. (2022). The translatability of genetic networks from model to crop species: lessons from the past and perspectives for the future. New Phytol. 236, 43–48. 10.1111/nph.18364 35801919

[B63] Ivanov I. E. Wright A. V. Cofsky J. C. Aris K. D. P. Doudna J. A. Bryant Z. (2020). Cas9 interrogates DNA in discrete steps modulated by mismatches and supercoiling, Proc. Natl. Acad. Sci. U. S. A. 117 **,** 5853–5860. 10.1073/pnas.1913445117 32123105 PMC7084090

[B64] Jia H. Xu J. Orbovic V. Zhang Y. Wang N. (2017). Editing citrus genome *via* SaCas9/sgRNA system. Front. Plant Sci. 8, 2135. 10.3389/fpls.2017.02135 29312390 PMC5732962

[B65] Jiang F. Zhou K. Ma L. Gressel S. Doudna J. A. (2015). STRUCTURAL BIOLOGY. A Cas9-guide RNA complex preorganized for target DNA recognition. Science 348, 1477–1481. 10.1126/science.aab1452 26113724

[B66] Jiang F. Taylor D. W. Chen J. S. Kornfeld J. E. Zhou K. Thompson A. J. (2016). Structures of a CRISPR-Cas9 R-loop complex primed for DNA cleavage. Science 351, 867–871. 10.1126/science.aad8282 26841432 PMC5111852

[B67] Jiang W. Feng S. Huang S. Yu W. Li G. Yang G. (2018). BE-PLUS: a new base editing tool with broadened editing window and enhanced fidelity. Cell Res. 28, 855–861. 10.1038/s41422-018-0052-4 29875396 PMC6082914

[B68] Jiang Y. Chai Y. Qiao D. Wang J. Xin C. Sun W. (2022). Optimized prime editing efficiently generates glyphosate-resistant rice plants carrying homozygous TAP-IVS mutation in EPSPS. Mol. Plant 15, 1646–1649. 10.1016/j.molp.2022.09.006 36101511

[B69] Jin S. Zong Y. Gao Q. Zhu Z. Wang Y. Qin P. (2019). Cytosine, but not adenine, base editors induce genome-wide off-target mutations in rice. Science 364, 292–295. 10.1126/science.aaw7166 30819931

[B70] Jin S. Fei H. Zhu Z. Luo Y. Liu J. Gao S. (2020). Rationally designed APOBEC3B cytosine base editors with improved specificity. Mol. Cell 79, 728–740. 10.1016/j.molcel.2020.07.005 32721385

[B71] Jinek M. Chylinski K. Fonfara I. Hauer M. Doudna J. A. Charpentier E. (2012). A programmable Dual-RNA-Guided DNA endonuclease in adaptive bacterial immunity. Science 337, 816–821. 10.1126/science.1225829 22745249 PMC6286148

[B72] Jinek M. Jiang F. Taylor D. W. Sternberg S. H. Kaya E. Ma E. (2014). Structures of Cas9 endonucleases reveal RNA-mediated conformational activation. Science 343, 1247997. 10.1126/science.1247997 24505130 PMC4184034

[B73] Kang B. C. Yun J. Y. Kim S. T. Shin Y. Ryu J. Choi M. (2018). Precision genome engineering through adenine base editing in plants. Nat. Plants 4, 427–431. 10.1038/s41477-018-0178-x 29867128

[B74] Kaya H. Mikami M. Endo A. Endo M. Toki S. (2016). Highly specific targeted mutagenesis in plants using *Staphylococcus aureus* Cas9. Sci. Rep. 6, 26871. 10.1038/srep26871 27226350 PMC4881040

[B75] Kim Y. B. Komor A. C. Levy J. M. Packer M. S. Zhao K. T. Liu D. R. (2017). Increasing the genome-targeting scope and precision of base editing with engineered Cas9-cytidine deaminase fusions. Nat. Biotechnol. 35, 371–376. 10.1038/nbt.3803 28191901 PMC5388574

[B76] Kim H. K. Yu G. Park J. Min S. Lee S. Yoon S. (2021). Predicting the efficiency of prime editing guide RNAs in human cells. Nat. Biotechnol. 39, 198–206. 10.1038/s41587-020-0677-y 32958957

[B77] Kim Y.-H. Kim N. Okafor I. Choi S. Min S. Lee J. (2023). Sniper2L is a high-fidelity Cas9 variant with high activity. Nat. Chem. Biol. 19, 972–980. 10.1038/s41589-023-01279-5 36894722 PMC10374439

[B78] Kleinstiver B. P. Prew M. S. Tsai S. Q. Nguyen N. T. Topkar V. V. Zheng Z. (2015a). Broadening the targeting range of *Staphylococcus aureus* CRISPR-Cas9 by modifying PAM recognition. Nat. Biotechnol. 33, 1293–1298. 10.1038/nbt.3404 26524662 PMC4689141

[B79] Kleinstiver B. P. Prew M. S. Tsai S. Q. Topkar V. V. Nguyen N. T. Zheng Z. (2015b). Engineered CRISPR-Cas9 nucleases with altered PAM specificities. Nature 523, 481–485. 10.1038/nature14592 26098369 PMC4540238

[B80] Kleinstiver B. P. Pattanayak V. Prew M. S. Tsai S. Q. Nguyen N. T. Zheng Z. (2016). High-fidelity CRISPR–Cas9 nucleases with no detectable genome-wide off-target effects. Nature 529, 490–495. 10.1038/nature16526 26735016 PMC4851738

[B81] Ko J. H. Son M. Y. Zhou Q. Molnarova L. Song L. Mlcouskova J. (2020). TREX2 exonuclease causes spontaneous mutations and stress-induced replication fork defects in cells expressing RAD51(K133A). Cell Rep. 33, 108543. 10.1016/j.celrep.2020.108543 33357432 PMC7896812

[B82] Koblan L. W. Doman J. L. Wilson C. Levy J. M. Tay T. Newby G. A. (2018). Improving cytidine and adenine base editors by expression optimization and ancestral reconstruction. Nat. Biotechnol. 36, 843–846. 10.1038/nbt.4172 29813047 PMC6126947

[B83] Kocak D. D. Josephs E. A. Bhandarkar V. Adkar S. S. Kwon J. B. Gersbach C. A. (2019). Increasing the specificity of CRISPR systems with engineered RNA secondary structures. Nat. Biotechnol. 37, 657–666. 10.1038/s41587-019-0095-1 30988504 PMC6626619

[B84] Komor A. C. Kim Y. B. Packer M. S. Zuris J. A. Liu D. R. (2016). Programmable editing of a target base in genomic DNA without double-stranded DNA cleavage. Nature 533, 420–424. 10.1038/nature17946 27096365 PMC4873371

[B85] Komor A. C. Zhao K. T. Packer M. S. Gaudelli N. M. Waterbury A. L. Koblan L. W. (2017). Improved base excision repair inhibition and bacteriophage Mu gam protein yields C:G-to-T:A base editors with higher efficiency and product purity. Sci. Adv. 3, eaao4774. 10.1126/sciadv.aao4774 28875174 PMC5576876

[B86] Kurt I. C. Zhou R. Iyer S. Garcia S. P. Miller B. R. Langner L. M. (2021). CRISPR C-to-G base editors for inducing targeted DNA transversions in human cells. Nat. Biotechnol. 39, 41–46. 10.1038/s41587-020-0609-x 32690971 PMC7854778

[B87] Labuhn M. Adams F. F. Ng M. Knoess S. Schambach A. Charpentier E. M. (2018). Refined sgRNA efficacy prediction improves large- and small-scale CRISPR-Cas9 applications. Nucleic Acids Res. 46, 1375–1385. 10.1093/nar/gkx1268 29267886 PMC5814880

[B88] Lazzarotto C. R. Malinin N. L. Li Y. Zhang R. Yang Y. Lee G. (2020). CHANGE-Seq reveals genetic and epigenetic effects on CRISPR–Cas9 genome-wide activity. Nat. Biotechnol. 38, 1317–1327. 10.1038/s41587-020-0555-7 32541958 PMC7652380

[B89] Lee J. K. Jeong E. Lee J. Jung M. Shin E. Kim Y.-H. (2018). Directed evolution of CRISPR-Cas9 to increase its specificity. Nat. Commun. 9, 3048. 10.1038/s41467-018-05477-x 30082838 PMC6078992

[B90] Lee H. K. Smith H. E. Liu C. Willi M. Hennighausen L. (2020). Cytosine base editor 4 but not adenine base editor generates off-target mutations in mouse embryos. Commun. Biol. 3, 19. 10.1038/s42003-019-0745-3 31925293 PMC6952419

[B91] Lee J. Lim K. Kim A. Mok Y. G. Chung E. Cho S. I. (2023). Prime editing with genuine Cas9 nickases minimizes unwanted indels. Nat. Commun. 14, 1786. 10.1038/s41467-023-37507-8 36997524 PMC10063541

[B92] Leenay R. t. Maksimchuk K. r. Slotkowski R. a. Agrawal R. n. Gomaa A. a. Beisel C. l. (2016). Identifying and visualizing functional PAM diversity across CRISPR-cas systems. Mol. Cell 62, 137–147. 10.1016/j.molcel.2016.02.031 27041224 PMC4826307

[B93] Li J. Vaidya M. White C. Vainstein A. Citovsky V. Tzfira T. (2005). Involvement of KU80 in T-DNA integration in plant cells. Proc. Natl. Acad. Sci. U. S. A. 102, 19231–19236. 10.1073/pnas.0506437103 16380432 PMC1323163

[B94] Li J. Meng X. Zong Y. Chen K. Zhang H. Liu J. (2016). Gene replacements and insertions in rice by intron targeting using CRISPR-Cas9. Nat. Plants 2, 16139. 10.1038/nplants.2016.139 27618611

[B95] Li C. Zong Y. Wang Y. Jin S. Zhang D. Song Q. (2018). Expanded base editing in rice and wheat using a Cas9-adenosine deaminase fusion. Genome Biol. 19, 59. 10.1186/s13059-018-1443-z 29807545 PMC5972399

[B96] Li C. Zong Y. Jin S. Zhu H. Lin D. Li S. (2020a). SWISS: multiplexed orthogonal genome editing in plants with a Cas9 nickase and engineered CRISPR RNA scaffolds. Genome Biol. 21, 141. 10.1186/s13059-020-02051-x 32546280 PMC7296638

[B97] Li H. Li J. Chen J. Yan L. Xia L. (2020b). Precise modifications of both exogenous and endogenous genes in rice by prime editing. Mol. Plant 13, 671–674. 10.1016/j.molp.2020.03.011 32222486

[B98] Li J. Xu R. Qin R. Liu X. Kong F. Wei P. (2021). Genome editing mediated by SpCas9 variants with broad non-canonical PAM compatibility in plants. Mol. Plant 14, 352–360. 10.1016/j.molp.2020.12.017 33383203

[B99] Li J. Chen L. Liang J. Xu R. Jiang Y. Li Y. (2022a). Development of a highly efficient prime editor 2 system in plants. Genome Biol. 23, 161. 10.1186/s13059-022-02730-x 35879771 PMC9310484

[B100] Li X. Wang X. Sun W. Huang S. Zhong M. Yao Y. (2022b). Enhancing prime editing efficiency by modified pegRNA with RNA G-quadruplexes. J. Mol. Cell Biol. 14, mjac022. 10.1093/jmcb/mjac022 35411929 PMC9345309

[B101] Li X. Zhou L. Gao B. Q. Li G. Wang X. Wang Y. (2022c). Highly efficient prime editing by introducing same-sense mutations in pegRNA or stabilizing its structure. Nat. Commun. 13, 1669. 10.1038/s41467-022-29339-9 35351879 PMC8964725

[B102] Li J. Ding J. Zhu J. Xu R. Gu D. Liu X. (2023). Prime editing-mediated precise knockin of protein tag sequences in the rice genome. Plant Commun. 4, 100572. 10.1016/j.xplc.2023.100572 36883004 PMC10203452

[B103] Liang Z. Wu Y. Guo Y. Wei S. (2023). Addition of the T5 exonuclease increases the prime editing efficiency in plants. J. Genet. Genomics 50, 582–588. 10.1016/j.jgg.2023.03.008 36958601

[B104] Lin Q. Zong Y. Xue C. Wang S. Jin S. Zhu Z. (2020). Prime genome editing in rice and wheat. Nat. Biotechnol. 38, 582–585. 10.1038/s41587-020-0455-x 32393904

[B105] Lin Q. Jin S. Zong Y. Yu H. Zhu Z. Liu G. (2021). High-efficiency prime editing with optimized, paired pegRNAs in plants. Nat. Biotechnol. 39, 923–927. 10.1038/s41587-021-00868-w 33767395

[B106] Liu X. Homma A. Sayadi J. Yang S. Ohashi J. Takumi T. (2016). Sequence features associated with the cleavage efficiency of CRISPR/Cas9 system. Sci. Rep. 6, 19675. 10.1038/srep19675 26813419 PMC4728555

[B107] Liu G. Yin K. Zhang Q. Gao C. Qiu J. L. (2019a). Modulating chromatin accessibility by transactivation and targeting proximal dsgRNAs enhances Cas9 editing efficiency *in vivo* . Genome Biol. 20, 145. 10.1186/s13059-019-1762-8 31349852 PMC6660936

[B108] Liu J. Nannas N. J. Fu F. F. Shi J. Aspinwall B. Parrott W. A. (2019b). Genome-scale sequence disruption following biolistic transformation in rice and maize. Plant Cell 31, 368–383. 10.1105/tpc.18.00613 30651345 PMC6447018

[B109] Liu Y. Yang G. Huang S. Li X. Wang X. Li G. (2021). Enhancing prime editing by Csy4-mediated processing of pegRNA. Cell Res. 31, 1134–1136. 10.1038/s41422-021-00520-x 34103663 PMC8486859

[B110] Liu B. Dong X. Cheng H. Zheng C. Chen Z. Rodriguez T. C. (2022). A split prime editor with untethered reverse transcriptase and circular RNA template. Nat. Biotechnol. 40, 1388–1393. 10.1038/s41587-022-01255-9 35379962

[B111] Liu D. Myers E. A. Xuan S. Prichard L. E. Donahue L. I. Ellison E. E. (2024a). Heritable, multinucleotide deletions in plants using viral delivery of a repair exonuclease and guide RNAs. Plant Physiol. 194, 2229–2239. 10.1093/plphys/kiae015 38243587

[B112] Liu M. Zhang X. Xu W. Kang G. Liu Y. Liu X. (2024b). Efficient and precise genomic deletion in rice using enhanced prime editing. aBIOTECH 5, 214–218. 10.1007/s42994-024-00153-9 38974869 PMC11224055

[B113] Liu X. Gu D. Zhang Y. Jiang Y. Xiao Z. Xu R. (2024c). Conditional knockdown of OsMLH1 to improve plant prime editing systems without disturbing fertility in rice. Genome Biol. 25, 131. 10.1186/s13059-024-03282-y 38773623 PMC11110357

[B114] Longo G. M. C. Sayols S. Kotini A. G. Heinen S. Mockel M. M. Beli P. (2024). Linking CRISPR-Cas9 double-strand break profiles to gene editing precision with BreakTag. Nat. Biotechnol. 43, 608–622. 10.1038/s41587-024-02238-8 38740992 PMC11994453

[B115] Lou H. Li S. Shi Z. Zou Y. Zhang Y. Huang X. (2025). Engineering source-sink relations by prime editing confers heat-stress resilience in tomato and rice. Cell 188, 530–549.e20. 10.1016/j.cell.2024.11.005 39674177

[B116] Lowe K. Wu E. Wang N. Hoerster G. Hastings C. Cho M. J. (2016). Morphogenic regulators baby boom and wuschel improve monocot transformation. Plant Cell 28, 1998–2015. 10.1105/tpc.16.00124 27600536 PMC5059793

[B117] Lu Y. Zhu J. K. (2017). Precise editing of a target base in the rice genome using a modified CRISPR/Cas9 system. Mol. Plant 10, 523–525. 10.1016/j.molp.2016.11.013 27932049

[B118] Lu Y. Tian Y. Shen R. Yao Q. Wang M. Chen M. (2020). Targeted, efficient sequence insertion and replacement in rice. Nat. Biotechnol. 38, 1402–1407. 10.1038/s41587-020-0581-5 32632302

[B119] Ma X. Zhang Q. Zhu Q. Liu W. Chen Y. Qiu R. (2015). A robust CRISPR/Cas9 system for convenient, high-efficiency multiplex genome editing in monocot and dicot plants. Mol. Plant 8, 1274–1284. 10.1016/j.molp.2015.04.007 25917172

[B120] Ma B. Wu H. Gou S. Lian M. Xia C. Yang K. (2024). A-to-G/C/T and C-to-T/G/A dual-function base editor for creating multi-nucleotide variants. J. Genet. Genomics 51, 1494–1504. 10.1016/j.jgg.2024.10.001 39490920

[B121] Malik A. Gul A. Munir F. Amir R. Alipour H. Babar M. M. (2021). Evaluating the cleavage efficacy of CRISPR-Cas9 sgRNAs targeting ineffective regions of *Arabidopsis thaliana* genome. PeerJ 9, e11409. 10.7717/peerj.11409 34055482 PMC8142926

[B122] Martinez-Galvez G. Joshi P. Friedberg I. Manduca A. Ekker S. C. (2021). Deploying MMEJ using MENdel in precision gene editing applications for gene therapy and functional genomics. Nucleic Acids Res. 49, 67–78. 10.1093/nar/gkaa1156 33305328 PMC7797032

[B123] Mekler V. Minakhin L. Severinov K. (2017). Mechanism of duplex DNA destabilization by RNA-guided Cas9 nuclease during target interrogation, Proc. Natl. Acad. Sci. U. S. A., 114 **,** 5443–5448. 10.1073/pnas.1619926114 28484024 PMC5448204

[B124] Miller S. M. Wang T. Randolph P. B. Arbab M. Shen M. W. Huang T. P. (2020). Continuous evolution of SpCas9 variants compatible with non-G PAMs. Nat. Biotechnol. 38, 471–481. 10.1038/s41587-020-0412-8 32042170 PMC7145744

[B125] Modrzejewski D. Hartung F. Lehnert H. Sprink T. Kohl C. Keilwagen J. (2020). Which factors affect the occurrence of off-target effects caused by the use of CRISPR/Cas: a systematic review in plants. Front. Plant Sci. 11, 574959. 10.3389/fpls.2020.574959 33329634 PMC7719684

[B126] Mojica F. J. M. Díez-Villaseñor C. García-Martínez J. Almendros C. (2009). Short motif sequences determine the targets of the prokaryotic CRISPR defence system. Microbiology 155, 733–740. 10.1099/mic.0.023960-0 19246744

[B127] Nelson J. W. Randolph P. B. Shen S. P. Everette K. A. Chen P. J. Anzalone A. V. (2022). Engineered pegRNAs improve prime editing efficiency. Nat. Biotechnol. 40, 402–410. 10.1038/s41587-021-01039-7 34608327 PMC8930418

[B128] Ni P. Zhao Y. Zhou X. Liu Z. Huang Z. Ni Z. (2023). Efficient and versatile multiplex prime editing in hexaploid wheat. Genome Biol. 24, 156. 10.1186/s13059-023-02990-1 37386475 PMC10308706

[B129] Nielsen S. Yuzenkova Y. Zenkin N. (2013). Mechanism of eukaryotic RNA polymerase III transcription termination. Science 340, 1577–1580. 10.1126/science.1237934 23812715 PMC3760304

[B130] Nishimasu H. Ran F. A. Hsu P. d. Konermann S. Shehata S. i. Dohmae N. (2014). Crystal structure of Cas9 in complex with guide RNA and target DNA. Cell 156, 935–949. 10.1016/j.cell.2014.02.001 24529477 PMC4139937

[B131] Nishimasu H. Shi X. Ishiguro S. Gao L. Hirano S. Okazaki S. (2018). Engineered CRISPR-Cas9 nuclease with expanded targeting space. Science 361, 1259–1262. 10.1126/science.aas9129 30166441 PMC6368452

[B132] Niu Q. Wu S. Xie H. Wu Q. Liu P. Xu Y. (2023). Efficient A·T to G·C base conversions in dicots using adenine base editors expressed under the tomato EF1α promoter. Plant Biotechnol. J. 21, 5–7. 10.1111/pbi.13736 34695289 PMC9829387

[B133] Organisms E. P. O. G. M. Mullins E. Bresson J. L. Dalmay T. Dewhurst I. C. Epstein M. M. (2024). Scientific opinion on the ANSES analysis of annex I of the EC proposal COM (2023) 411 (EFSA-Q-2024-00178). EFSA J. 22, e8894. 10.2903/j.efsa.2024.8894 38993591 PMC11237874

[B134] Pacesa M. Lin C.-H. Cléry A. Saha A. Arantes P. R. Bargsten K. (2022a). Structural basis for Cas9 off-target activity. Cell 185, 4067–4081.e21. 10.1016/j.cell.2022.09.026 36306733 PMC10103147

[B135] Pacesa M. Loeff L. Querques I. Muckenfuss L. M. Sawicka M. Jinek M. (2022b). R-loop formation and conformational activation mechanisms of Cas9. Nature 609, 191–196. 10.1038/s41586-022-05114-0 36002571 PMC9433323

[B136] Palermo G. Chen J. S. Ricci C. G. Rivalta I. Jinek M. Batista V. S. (2018). Key role of the REC lobe during CRISPR–Cas9 activation by ‘sensing’, ‘regulating’, and ‘locking’ the catalytic HNH domain. Q. Rev. Biophysics 51, e91. 10.1017/S0033583518000070 30555184 PMC6292676

[B137] Paliksa S. Alzbutas G. Skirgaila R. (2018). Decreased Km to dNTPs is an essential M-MuLV reverse transcriptase adoption required to perform efficient cDNA synthesis in one-step RT-PCR assay. Protein Eng. Des. Sel. 31, 79–89. 10.1093/protein/gzy003 29608777

[B138] Ponnienselvan K. Liu P. Nyalile T. Oikemus S. Maitland S. A. Lawson N. D. (2023). Reducing the inherent auto-inhibitory interaction within the pegRNA enhances prime editing efficiency. Nucleic Acids Res. 51, 6966–6980. 10.1093/nar/gkad456 37246708 PMC10359601

[B139] Qi Y. Zhang Y. Zhang F. Baller J. A. Cleland S. C. Ryu Y. (2013). Increasing frequencies of site-specific mutagenesis and gene targeting in arabidopsis by manipulating DNA repair pathways. Genome Res. 23, 547–554. 10.1101/gr.145557.112 23282329 PMC3589543

[B140] Qiao D. Wang J. Lu M. H. Xin C. Chai Y. Jiang Y. (2023). Optimized prime editing efficiently generates heritable mutations in maize. J. Integr. Plant Biol. 65, 900–906. 10.1111/jipb.13428 36478403

[B141] Qin R. Li J. Li H. Zhang Y. Liu X. Miao Y. (2019). Developing a highly efficient and wildly adaptive CRISPR-SaCas9 toolset for plant genome editing. Plant Biotechnol. J. 17, 706–708. 10.1111/pbi.13047 30537191 PMC6419570

[B142] Raitskin O. Schudoma C. West A. Patron N. J. (2019). Comparison of efficiency and specificity of CRISPR-associated (cas) nucleases in plants: an expanded toolkit for precision genome engineering. PLoS One 14, e0211598. 10.1371/journal.pone.0211598 30811422 PMC6392405

[B143] Riesenberg S. Helmbrecht N. Kanis P. Maricic T. Pääbo S. (2022). Improved gRNA secondary structures allow editing of target sites resistant to CRISPR-Cas9 cleavage. Nat. Commun. 13, 489. 10.1038/s41467-022-28137-7 35078986 PMC8789806

[B144] Rose J. C. Popp N. A. Richardson C. D. Stephany J. J. Mathieu J. Wei C. T. (2020). Suppression of unwanted CRISPR-Cas9 editing by co-administration of catalytically inactivating truncated guide RNAs. Nat. Commun. 11, 2697. 10.1038/s41467-020-16542-9 32483117 PMC7264211

[B145] Safari F. Zare K. Negahdaripour M. Barekati-Mowahed M. Ghasemi Y. (2019). CRISPR Cpf1 proteins: structure, function and implications for genome editing. Cell and Biosci. 9, 36. 10.1186/s13578-019-0298-7 31086658 PMC6507119

[B146] Saika H. Nishizawa-Yokoi A. Toki S. (2014). The non-homologous end-joining pathway is involved in stable transformation in rice. Front. Plant Sci. 5, 560. 10.3389/fpls.2014.00560 25368624 PMC4201092

[B147] Shah S. A. Erdmann S. Mojica F. J. M. Garrett R. A. (2013). Protospacer recognition motifs: mixed identities and functional diversity. RNA Biol. 10, 891–899. 10.4161/rna.23764 23403393 PMC3737346

[B148] Shen L. Wang C. Fu Y. Wang J. Liu Q. Zhang X. (2018). QTL editing confers opposing yield performance in different rice varieties. J. Integr. Plant Biol. 60, 89–93. 10.1111/jipb.12501 27628577

[B149] Spencer J. M. Zhang X. (2017). Deep mutational scanning of S. pyogenes Cas9 reveals important functional domains. Sci. Rep. 7, 16836. 10.1038/s41598-017-17081-y 29203891 PMC5715146

[B150] Sretenovic S. Liu S. Li G. Cheng Y. Fan T. Xu Y. (2021a). Exploring C-To-G base editing in rice, tomato, and poplar. Front. Genome 3, 756766. 10.3389/fgeed.2021.756766 34713268 PMC8525388

[B151] Sretenovic S. Yin D. Levav A. Selengut J. D. Mount S. M. Qi Y. (2021b). Expanding plant genome-editing scope by an engineered iSpyMacCas9 system that targets A-rich PAM sequences. Plant Commun. 2, 100101. 10.1016/j.xplc.2020.100101 33898973 PMC8060698

[B152] Steinert J. Schiml S. Fauser F. Puchta H. (2015). Highly efficient heritable plant genome engineering using Cas9 orthologues from Streptococcus thermophilus and *Staphylococcus aureus* . Plant J. 84, 1295–1305. 10.1111/tpj.13078 26576927

[B153] Sternberg S. H. Lafrance B. Kaplan M. Doudna J. A. (2015). Conformational control of DNA target cleavage by CRISPR–Cas9. Nature 527, 110–113. 10.1038/nature15544 26524520 PMC4859810

[B154] Sun C. Lei Y. Li B. Gao Q. Li Y. Cao W. (2024). Precise integration of large DNA sequences in plant genomes using PrimeRoot editors. Nat. Biotechnol. 42, 316–327. 10.1038/s41587-023-01769-w 37095350

[B155] Szczelkun M. D. Tikhomirova M. S. Sinkunas T. Gasiunas G. Karvelis T. Pschera P. (2014). Direct observation of R-loop formation by single RNA-guided Cas9 and Cascade effector complexes. Proc. Natl. Acad. Sci. 111, 9798–9803. 10.1073/pnas.1402597111 24912165 PMC4103346

[B156] Tang X. Zheng X. Qi Y. Zhang D. Cheng Y. Tang A. (2016). A single transcript CRISPR-Cas9 system for efficient genome editing in plants. Mol. Plant 9, 1088–1091. 10.1016/j.molp.2016.05.001 27212389

[B157] Tian Y. Shen R. Li Z. Yao Q. Zhang X. Zhong D. (2022). Efficient C-to-G editing in rice using an optimized base editor. Plant Biotechnol. J. 20, 1238–1240. 10.1111/pbi.13841 35534986 PMC9241366

[B158] Truong D. J. Geilenkeuser J. Wendel S. V. Wilming J. C. H. Armbrust N. Binder E. M. H. (2024). Exonuclease-enhanced prime editors. Nat. Methods 21, 455–464. 10.1038/s41592-023-02162-w 38302659 PMC10927552

[B159] Tsai S. Q. Zheng Z. Nguyen N. T. Liebers M. Topkar V. V. Thapar V. (2015). GUIDE-seq enables genome-wide profiling of off-target cleavage by CRISPR-cas nucleases. Nat. Biotechnol. 33, 187–197. 10.1038/nbt.3117 25513782 PMC4320685

[B160] Vakulskas C. A. Dever D. P. Rettig G. R. Turk R. Jacobi A. M. Collingwood M. A. (2018). A high-fidelity Cas9 mutant delivered as a ribonucleoprotein complex enables efficient gene editing in human hematopoietic stem and progenitor cells. Nat. Med. 24, 1216–1224. 10.1038/s41591-018-0137-0 30082871 PMC6107069

[B161] Vu T. V. Nguyen N. T. Kim J. Das S. Lee J. Kim J. Y. (2022). The obstacles and potential solution clues of prime editing applications in tomato. Biodes Res. 2022, 0001. 10.34133/bdr.0001 37905201 PMC10593121

[B162] Vu T. V. Nguyen N. T. Kim J. Song Y. J. Nguyen T. H. Kim J. Y. (2024). Optimized dicot prime editing enables heritable desired edits in tomato and arabidopsis. Nat. Plants 10, 1502–1513. 10.1038/s41477-024-01786-w 39242983

[B163] Walton R. T. Christie K. A. Whittaker M. N. Kleinstiver B. P. (2020). Unconstrained genome targeting with near-PAMless engineered CRISPR-Cas9 variants. Science 368, 290–296. 10.1126/science.aba8853 32217751 PMC7297043

[B164] Waltz E. (2022). GABA-enriched tomato is first CRISPR-edited food to enter market. Nat. Biotechnol. 40, 9–11. 10.1038/d41587-021-00026-2 34907351

[B165] Wang X. Li J. Wang Y. Yang B. Wei J. Wu J. (2018). Efficient base editing in methylated regions with a human APOBEC3A-Cas9 fusion. Nat. Biotechnol. 36, 946–949. 10.1038/nbt.4198 30125268

[B166] Wang D. Zhang C. Wang B. Li B. Wang Q. Liu D. (2019). Optimized CRISPR guide RNA design for two high-fidelity Cas9 variants by deep learning. Nat. Commun. 10, 4284. 10.1038/s41467-019-12281-8 31537810 PMC6753114

[B167] Wang Q. Bian X. Du J. Lv Y. Tao L. Xie T. (2021). PAM-interacting domain swapping is extensively utilized in nature to evolve CRISPR-Cas9 nucleases with altered PAM specificities. BioRxiv. 10.1101/2021.05.01.442224

[B168] Wang J. He Z. Wang G. Zhang R. Duan J. Gao P. (2022a). Efficient targeted insertion of large DNA fragments without DNA donors. Nat. Methods 19, 331–340. 10.1038/s41592-022-01399-1 35228726

[B169] Wang W. Wang W. Pan Y. Tan C. Li H. Chen Y. (2022b). A new gain-of-function OsGS2/GRF4 allele generated by CRISPR/Cas9 genome editing increases rice grain size and yield. Crop J. 10, 1207–1212. 10.1016/j.cj.2022.01.004

[B170] Wang P. Si H. Li C. Xu Z. Guo H. Jin S. (2025). Plant genetic transformation: achievements, current status and future prospects. Plant Biotechnol. J. 23, 2034–2058. 10.1111/pbi.70028 40052992 PMC12120897

[B171] Xie K. Minkenberg B. Yang Y. (2015). Boosting CRISPR/Cas9 multiplex editing capability with the endogenous tRNA-processing system. Proc. Natl. Acad. Sci. U. S. A. 112, 3570–3575. 10.1073/pnas.1420294112 25733849 PMC4371917

[B172] Xie H. Ge X. Yang F. Wang B. Li S. Duan J. (2020). High-fidelity SaCas9 identified by directional screening in human cells. PLoS Biol. 18, e3000747. 10.1371/journal.pbio.3000747 32644995 PMC7347106

[B173] Xu K. Xu X. Fukao T. Canlas P. Maghirang-Rodriguez R. Heuer S. (2006). Sub1A is an ethylene-response-factor-like gene that confers submergence tolerance to rice. Nature 442, 705–708. 10.1038/nature04920 16900200

[B174] Xu W. Song W. Yang Y. Wu Y. Lv X. Yuan S. (2019). Multiplex nucleotide editing by high-fidelity Cas9 variants with improved efficiency in rice. BMC Plant Biol. 19, 511. 10.1186/s12870-019-2131-1 31752697 PMC6873407

[B175] Xu R. Li J. Liu X. Shan T. Qin R. Wei P. (2020a). Development of plant prime-editing systems for precise genome editing. Plant Commun. 1, 100043. 10.1016/j.xplc.2020.100043 33367239 PMC7747961

[B176] Xu Y. Meng X. Wang J. Qin B. Wang K. Li J. (2020b). ScCas9 recognizes NNG protospacer adjacent motif in genome editing of rice. Sci. China Life Sci. 63, 450–452. 10.1007/s11427-019-1630-2 31953707

[B177] Xu Y. Wang F. Chen Z. Wang J. Li W.-Q. Fan F. (2020c). Intron-targeted gene insertion in rice using CRISPR/Cas9: a case study of the Pi-ta gene. Crop J. 8, 424–431. 10.1016/j.cj.2019.03.006

[B178] Xu Z. Kuang Y. Ren B. Yan D. Yan F. Spetz C. (2021). SpRY greatly expands the genome editing scope in rice with highly flexible PAM recognition. Genome Biol. 22, 6. 10.1186/s13059-020-02231-9 33397431 PMC7780387

[B179] Xu R. Qin R. Xie H. Li J. Liu X. Zhu M. (2022a). Genome editing with type II-C CRISPR-Cas9 systems from Neisseria meningitidis in rice. Plant Biotechnol. J. 20, 350–359. 10.1111/pbi.13716 34582079 PMC8753361

[B180] Xu W. Yang Y. Yang B. Krueger C. J. Xiao Q. Zhao S. (2022b). A design optimized prime editor with expanded scope and capability in plants. Nat. Plants 8, 45–52. 10.1038/s41477-021-01043-4 34949802

[B181] Xu R. Ma C. Sheng J. Zhu J. Wang D. Liu X. (2024). Engineering PE6 prime editors to efficiently insert tags in rice. Plant Biotechnol. J. 22, 3383–3385. 10.1111/pbi.14456 39331467 PMC11606421

[B182] Yamamoto A. Ishida T. Yoshimura M. Kimura Y. Sawa S. (2019). Developing heritable mutations in Arabidopsis thaliana using a modified CRISPR/Cas9 toolkit comprising PAM-altered Cas9 variants and gRNAs. Plant Cell Physiol. 60, 2255–2262. 10.1093/pcp/pcz118 31198958

[B183] Yang L. Machin F. Wang S. Saplaoura E. Kragler F. (2023). Heritable transgene-free genome editing in plants by grafting of wild-type shoots to transgenic donor rootstocks. Nat. Biotechnol. 41, 958–967. 10.1038/s41587-022-01585-8 36593415 PMC10344777

[B184] Yarnall M. T. N. Ioannidi E. I. Schmitt-Ulms C. Krajeski R. N. Lim J. Villiger L. (2023). Drag-and-drop genome insertion of large sequences without double-strand DNA cleavage using CRISPR-directed integrases. Nat. Biotechnol. 41, 500–512. 10.1038/s41587-022-01527-4 36424489 PMC10257351

[B185] Ye L. Wang C. Hong L. Sun N. Chen D. Chen S. (2018). Programmable DNA repair with CRISPRa/i enhanced homology-directed repair efficiency with a single Cas9. Cell Discov. 4, 46. 10.1038/s41421-018-0049-7 30062046 PMC6056518

[B186] Yin J. Lu R. Xin C. Wang Y. Ling X. Li D. (2022). Cas9 exo-endonuclease eliminates chromosomal translocations during genome editing. Nat. Commun. 13, 1204. 10.1038/s41467-022-28900-w 35260581 PMC8904484

[B187] Yuen G. Khan F. J. Gao S. Stommel J. M. Batchelor E. Wu X. (2017). CRISPR/Cas9-mediated gene knockout is insensitive to target copy number but is dependent on guide RNA potency and Cas9/sgRNA threshold expression level. Nucleic Acids Res. 45, 12039–12053. 10.1093/nar/gkx843 29036671 PMC5714203

[B188] Zeng D. Li X. Huang J. Li Y. Cai S. Yu W. (2020). Engineered Cas9 variant tools expand targeting scope of genome and base editing in rice. Plant Biotechnol. J. 18, 1348–1350. 10.1111/pbi.13293 31696609 PMC7206991

[B189] Zetsche B. Gootenberg J. s. Abudayyeh O. o. Slaymaker I. m. Makarova K. s. Essletzbichler P. (2015). Cpf1 is a single RNA-guided endonuclease of a class 2 CRISPR-cas system. Cell 163, 759–771. 10.1016/j.cell.2015.09.038 26422227 PMC4638220

[B190] Zhang Q. Wen F. Zhang S. Jin J. Bi L. Lu Y. (2019). The post-PAM interaction of RNA-guided spCas9 with DNA dictates its target binding and dissociation. Sci. Adv. 5, eaaw9807. 10.1126/sciadv.aaw9807 31763447 PMC6853773

[B191] Zhang C. Xu W. Wang F. Kang G. Yuan S. Lv X. (2020). Expanding the base editing scope to GA and relaxed NG PAM sites by improved xCas9 system. Plant Biotechnol. J. 18, 884–886. 10.1111/pbi.13259 31545544 PMC7061872

[B192] Zhang Y. Ren Q. Tang X. Liu S. Malzahn A. A. Zhou J. (2021). Expanding the scope of plant genome engineering with Cas12a orthologs and highly multiplexable editing systems. Nat. Commun. 12, 1944. 10.1038/s41467-021-22330-w 33782402 PMC8007695

[B193] Zhang Y. Cai Y. Sun S. Han T. Chen L. Hou W. (2022). Using Staphylococcus aureus Cas9 to expand the scope of potential gene targets for genome editing in soybean. Int. J. Mol. Sci. 23, 12789. 10.3390/ijms232112789 36361580 PMC9658631

[B194] Zhang H. Ma J. Wu Z. Chen X. Qian Y. Chen W. (2024). BacPE: a versatile prime-editing platform in bacteria by inhibiting DNA exonucleases. Nat. Commun. 15, 825. 10.1038/s41467-024-45114-4 38280845 PMC10821919

[B195] Zheng C. Liu B. Dong X. Gaston N. Sontheimer E. J. Xue W. (2023). Template-jumping prime editing enables large insertion and exon rewriting *in vivo* . Nat. Commun. 14, 3369. 10.1038/s41467-023-39137-6 37291100 PMC10250319

[B196] Zhong Z. Sretenovic S. Ren Q. Yang L. Bao Y. Qi C. (2019). Improving plant genome editing with high-fidelity xCas9 and non-canonical PAM-targeting Cas9-NG. Mol. Plant 12, 1027–1036. 10.1016/j.molp.2019.03.011 30928637

[B197] Zhong Z. Fan T. He Y. Liu S. Zheng X. Xu Y. (2024). An improved plant prime editor for efficient generation of multiple-nucleotide variations and structural variations in rice. Plant Commun. 5, 100976. 10.1016/j.xplc.2024.100976 38751122 PMC11412927

[B198] Zhou J. Xin X. He Y. Chen H. Li Q. Tang X. (2019). Multiplex QTL editing of grain-related genes improves yield in elite rice varieties. Plant Cell Rep. 38, 475–485. 10.1007/s00299-018-2340-3 30159598

[B199] Zhou S. Cai L. Wu H. Wang B. Gu B. Cui S. (2024). Fine-tuning rice heading date through multiplex editing of the regulatory regions of key genes by CRISPR-Cas9. Plant Biotechnol. J. 22, 751–758. 10.1111/pbi.14221 37932934 PMC10893950

[B200] Zong Y. Wang Y. Li C. Zhang R. Chen K. Ran Y. (2017). Precise base editing in rice, wheat and maize with a Cas9-cytidine deaminase fusion. Nat. Biotechnol. 35, 438–440. 10.1038/nbt.3811 28244994

[B201] Zong Y. Liu Y. Xue C. Li B. Li X. Wang Y. (2022). An engineered prime editor with enhanced editing efficiency in plants. Nat. Biotechnol. 40, 1394–1402. 10.1038/s41587-022-01254-w 35332341

[B202] Zuo Z. Liu J. (2017). Structure and dynamics of Cas9 HNH domain catalytic state. Sci. Rep. 7, 17271. 10.1038/s41598-017-17578-6 29222528 PMC5722908

[B203] Zuo E. Sun Y. Wei W. Yuan T. Ying W. Sun H. (2019). Cytosine base editor generates substantial off-target single-nucleotide variants in mouse embryos. Science 364, 289–292. 10.1126/science.aav9973 30819928 PMC7301308

